# The Genus *Solanum*: An Ethnopharmacological, Phytochemical and Biological Properties Review

**DOI:** 10.1007/s13659-019-0201-6

**Published:** 2019-03-12

**Authors:** Joseph Sakah Kaunda, Ying-Jun Zhang

**Affiliations:** 1 State Key Laboratory of Phytochemistry and Plant Resources in West China, Kunming Institute of Botany, Chinese Academy of Sciences, Kunming, 650204 People’s Republic of China; 2 Graduate School of the Chinese Academy of Sciences, Beijing, 100039 People’s Republic of China; 3 Yunnan Key Laboratory of Natural Medicinal Chemistry, Kunming Institute of Botany, Chinese Academy of Sciences, Kunming, 650201 People’s Republic of China

**Keywords:** *Solanum*, Solanaceae, Phytochemistry, Steroidal saponins and alkaloids, Ethnopharmacology

## Abstract

Over the past 30 years, the genus *Solanum* has received considerable attention in chemical and biological studies*. Solanum* is the largest genus in the family Solanaceae, comprising of about 2000 species distributed in the subtropical and tropical regions of Africa, Australia, and parts of Asia, e.g., China, India and Japan. Many of them are economically significant species. Previous phytochemical investigations on *Solanum* species led to the identification of steroidal saponins, steroidal alkaloids, terpenes, flavonoids, lignans, sterols, phenolic comopunds, coumarins, amongst other compounds. Many species belonging to this genus present huge range of pharmacological activities such as cytotoxicity to different tumors as breast cancer (4T1 and EMT), colorectal cancer (HCT116, HT29, and SW480), and prostate cancer (DU145) cell lines. The biological activities have been attributed to a number of steroidal saponins, steroidal alkaloids and phenols. This review features 65 phytochemically studied species of *Solanum* between 1990 and 2018, fetched from SciFinder, Pubmed, ScienceDirect, Wikipedia and Baidu, using “Solanum” and the species’ names as search terms (“all fields”).

## Introduction

The genus *Solanum* is considered to be one of the largest and most complex genera among the Angiosperms [1], and the most representative and largest genus of the family Solanaceae [1–4]. It is comprised of about 2000 species distributed across subtropical and tropical regions of Asia [3–9], tropical Africa [10–29], non-arid Africa [30–43], Americas [44–87], Australia [71–74, 81–84] and India [71]. The genus is well represented in Brazil with about 350 species widely distributed from north to south in diverse phytogeographic regions [70, 80]. In Brazil (Ceará, Bahia, Mato Grosso do Sul, Paraná and north-central coast of Santa Catarina State), many *Solanum* species, usually known as ‘yubeba’, the word that refers to the prickles found on the stems of several of the species, are widely used in traditional medicine [66, 80, 87]. In the northeast of Brazil, 80 *Solanum* species are distributed throughout the region and used in folk medicine. One of such species is *S. capsicoides*, commonly known as “Gogoia” [87]. In East Africa, several *Solanum* species such as *S. arundo* and *S. incanum* are known to be poisonous and are reportedly used to induce miscarriages [64].

*Solanum* genus is rich in economically significant species; the food crops include *S. aethiopicum* [20, 21], *S. anguivi* [30, 31] *S. lycopersicum*, *S. melongena*, *S. muricatum*, *S. torvum* and *S. tuberosum* [1]. Ornamental species include *S. aviculare*, *S. capsicastrum*, *S. crispum*, *S. laciniatum*, *S. laxum*, *S. pseudocapsicum*, *S. rantonnetii*, *S. seaforthianum* and *S. wendlandii* [1].

A series of pharmacological studies have been carried out to verify and validate the traditional medicinal applications of many plants in this genus. The studied pharmacological activities include analgesic, anthelminthic, antiallergic, anti-anemic, anti-asthmatic, antibacterial, anti- cancer, anti-convulsant, anti-depressant, anti-diabetic, anti-fungal, antihistaminic, antihyperten- sive, anti-inflammatory, anti-leishmanial, antimelanogenetic, anti-molluscicidal, anti-nociceptive, anti-psoriatic, antiplasmodial, antiprotozoa, anti-trypanosomal, antiurolithiatic, antiviral, cardio- vascular, diuretic, hepatoprotective, hypolipidemic, mosquito larvicidal, nephrotoxic, spasmolytic, schistosomicidal and vasorelaxant activities.

In the past, several reviews on *Solanum* genus have been documented [88–101], however, mostly with singular focus on particular species. The present review is multi faceted, and features 66 medicinal species of *Solanum* in their geographical distribution, traditional uses, and 670 isolated chemical constituents, including 134 steroidal saponins, 63 steroidal alkaloids, 13 pregnane glycosides, 128 terpenes, 75 flavonoids, 31 lignans, 31 other types of alkaloids, 66 sterols, 52 phenolic compounds, 20 coumarins and coumestans, 4 coumarinolignoids, 23 fatty acids and esters and 30 other compounds. Where applicable, the biological activities of compounds isolated from various species are noted.

## Distribution and Ethnopharmacological Uses

Sixty-six species commonly used as important folk medicine, ornamental plants, or wild food sources were selected in this review, and their local names, distribution and ethnopharmacologi- cal uses were summarized in Table 1. Local names are given in different languages with which the inhabitants of a particular region use to identify a specific species. Each species’ natural habitat and/or places of cultivation are mentioned. Traditional as well as modern day applications are presented.

**Table 1:** Distribution and ethnopharmalogical uses of *Solanum* species

No.	Species	Local names	Distribution	Uses
1	*S*. *abutiloides*	Dwarf tamarillo	Argentina, Bolivia [2, 3]	Ornamental, fruits edible, anti-fungal [2–4]
2	*S. aculeastrum*	Goat bitter/poison/gifa/bok-bitter -apple, thola, murulwa, umthuma, itunga, mtuma	Kenya, South Africa, Swaziland [10]	Toothache, ringworm [10], jigger wounds, gonorrhea, anti-molluscicidal [11, 12], anticancer [13–15], antifungal [16], antimicrobial [12, 17], anti-leishmanial [18]
3	*S. aethiopicum*	African scarlet/Ethiopian/Chinese scarlet/tomato-fruit eggplant, azoko, garden egg, gilo, golden/love apple, impwa, kumba, losuke, mock/bitter/ruffed tomato, nakasuga, nakati, ngogwe, osun, tokalu, african aubergine, aubergine amère, Ethiopian nightshade, gilo, granadillo, jilo, kumba, meloncillo de olor, meloncillo del campo, pocotillo, quillo, revienta caballo, röd aubergin, shum, silverleaf nightshade, tutía enano	China, India, Japan, Angola, Benin, Botswana,Burkina Faso, Burundi, Cameroon, Cape Verde, Central Africa, Chad, Comoros, Congo DR, Djibouti, Egypt, Equatorial Guinea, Eritrea, Ethiopia, Gabon, Gambia, Ghana, Guinea, Guinea-Bissau, Ivory Coast, Liberia, Madagascar, Malawi, Mali, Mauritania, Mauritius, Mozambique, Namibia, Niger, Nigeria, Rwanda, Senegal, Sierra Leone, Sudan, Togo, Zambia, Zimbabwe, Australia, Brazil, Italy, France [20, 21]	Fruits/leaves eaten, ornamental [20, 21], anti-ulcer, anticancer [23–26], anti-inflammatory [27]
4	*S. agrarium*	Gogóia (Brazil)	Brazil, Guyana, Venezuela [44]	Mycosis, diarrhea, gonorrhea, prostatic, inflammation, abortion [44, 45]
5	*S. americanum*	American black/white/small flower/glossy nightshade, maria pretinha (Brazil), quilete (Guatemala), popolo (Hawaii)	Tropical Pacific, Indian Ocean, Hawaii, Indochina, Brazil, Madagascar, Africa	Ripe fruit makes jams, preservative, shoots eaten, antiviral, antimicrobial [46, 47], antidiabetic [48, 49], bladder spasm, joint pains, cooling, cough, gastric ulcer, protozoal infections, vermifuge [49], anticancer [47, 50–52], asthma [53]
6	*S. amygdalifolium*		Uruguay, Argentina, Brazil	Decoration [56]
7	*S. anguivi*	Forest bitterberry, African eggplant	Non-arid Africa: Nigeria, Ghana	Leaves/fruits consumed, coughs, dysuria, nasal ulcers, asthma, toothache, cardiac disorder, worm complaints, spinal chord and nervous disorder, fever, diabetes, artherosclerosis carminative, nasal ulcers, asthma, parturition, worm expeller, itching [30–32], hypolipidemic [33, 34], anaemia [31, 32, 35], Huntington’s, Alzheimer, Parkinson, amyotrophic lateral sclerosis [36], antioxidant [33, 37–39], hypotensive [38]
8	*S. arboreum*		Costa Rica, Colombia, Trinidad	Anti-leishmanial [60, 61], antimalarial [62]
9	*S. arundo*		Kenya	Abortion [64], hepatoprotective [65]
10	*S. asperum*		Brazil	Anti-molluscicidal [66], antifungal [67]
11	*S. asterophorum*	Jurubeba-de-fogo	Brazil	Liver dysfunctions, antidiarrheal [68], spasmolytic [69]
12	*S. betaceum*	English: tree tomato, South America: tamamoro and tomate de árbol, French: arbre à tomates, tomate de La Paz, tomate en arbre. Spanish: tamarillo, tomate de árbol, tomate Serrano	Ecuador, Colombia, Peru, Bolivia, Rwanda, South Africa, India, Nepal China, United States, Chile, Australia, New Zealand, Malaysia, Philippines, Puerto Rico, Bhutan [71–74]	Ripe fruit edible, preservative [71, 72], antioxidant [75]
13	*S. buddleifolium*	Unknown	Brazil [79]	Unknown
14	*S. caavurana*	Laranjinha do mato, ‘jurubebarana’ or ‘jurubeba-branca’	Brazil (Ceará, Bahia, Mato Grosso do Sul, Paraná,Santa Catarina States), Paraguay, Argentina	Anemia, liver disorders, digestion [80]
15	*S. capsicoides*	Cockroach berry, polohauai’i (Polynesia), devil’s apple	Brazil, Central America, Australia, Brooklyn, New York [81–84]	Ornamental [83], anti-inflammatory [85], anticancer [86], antihypertensive [87]
16	*S. cathayanum*		China	Anti-inflammatory, anti-bacterial [102], antitumor, anti-neurodegenerative [102–106]
17	S. cernuum	“Panaceia”	Brazil	Gastric ulcers, hepatic injuries, skin disorders, anti-tumor, depurative, diuretic, antihemorrhagic, antiblennorrhoea, cardiac disorders, analgesic, anti-inflammatory, urinary disorders, gastric cancer, gonorrhea [107–112]
18	*S. chrysotrichum*	“Sosa”	Mexico	Anti-mycotic, anti-inflammatory [113–120]
19	*S. cornifolium*		Latin America	Anti-mycotic [121]
20	*S. crinitum*	“jurubeba” and “fruto-de-lobo”	Brazil, Colombia	Anti-tumor [122, 123]
21	*S. diphyllum*		Mexico, Belize, Costa Rica, El Salvador, Guatemala, Honduras, Nicaragua, Florida, Texas, Indonesia, Philippines, West Indies, China, Taiwan, Egypt [124–126]	Anti-tumor [126]
22	*S. dulcamara*	Bittersweet/bitter/European/deadly/blue climbing/woody nightshade, felonwort, violet-bloom, fellen, scarlet/snake berry, mortal, fever twig, staff vine	Northern Africa, North America, Europe, Asia	Skin diseases, cancers, anti-tumors, alterative, anodyne, depurative, mildly diuretic, emetic, expectorant, hepatic, mildly narcotic and purgative [127–131], skin abrasions, inflammation [132]
23	*S. elaeagnifolium*	Prairie berry, Silverleaf nightshade, silverleaf/Whitehorse/bull/horse nettle (English); silver-leaf bitter-apple, Satan’s bush (South Africa); trompillo (Spanish); meloncillo del campo quillo-quillo, revienta caballo (Argentina); tomatillo (Chile); trompillo (Honduras)[540]	Mexico, USA, South America, Middle East, Southern Africa, North Africa, Taiwan, Penghu Islands, Brazil, India, Germany, Kenya [539, 540]	Contraceptive, corticosteroid drugs, hepatoprotective, hypoglycemic, hepatotonic, laxative, appetizer, cardiotonic, antispasmodic, antiepileptic, renal pain, analgesic, anti-inflammatory, anticancer, antimolluscicidal [133, 134]
24	*S. erianthum*	Aamourette marron (French); big eggplant, black/mullein nightshade, China flowerleaf, flannel bush, tropillo, turkey berry, wild tobacco, jia yan ye shu (Chinese)	Americas, Cuba, Dominican Republic, Haiti, Jamaica, Trinidad, South America	Leukorrhea, abortion, analgesic, vertigo, dysentery, fever, diarrhea, digestive problems, anti-inflammatory, leprosy, sexually-transmitted diseases, malaria, laxative, anti-diuretic, antihepatitis B, anti-tumor [135–139]
25	*S. glabratum*		Saudi Arabia, Yemen	Antibacteria, diuretic, scabies, syphilis, cough, hemorrhoids, anticancer [140–144]
26	*S. glaucophyllum*		Brazil, Bolivia, Argentina, Paraguay, Uruguay	Anticancer [145, 146]
27	*S. guaraniticum*	Jurubeba, false-jurubeba	Brazil, Paraguay, Argentina	Anemia, fevers, erysipela, hepatitis, ulcers, uterine tumors, tonic, digestive stimulant, fevers, antioxidants [147–149]
28	*S. incanum*	Thorn/bitter/sodom/poison/snake apple, mutongu (Kikuyu), mtunguja mwitu (Kiswahili), ochok (Luo)	Kenya, Uganda,Tanzania, Middle East, India, Australia, Madagascar, Mauritius, Saudi Arabia [150, 151]	Antibacterial [152, 153], antileishmanial [154], anticancer [155] conjunctivitis, inflammations [156]
29	*S. indicum*	Poison berry, Indian nightshade, African eggplant, bush tomato, ntunfulu, bhantaki, bari kateri, kateli, kshudra bhantaaki, mahati, mahotika, vartaki, vrihati, kataai kalaan, mullamkatti, papparamulli, barahantaa	India, Sri Lanka, Malaysia, China, Philippine Islands, Africa [157–159]	Diaphoretic, diuretic, expectorant, stimulant, bronchites, itching, bodyaches, asthma, wounds, toothache, narcotic, cutaneous disorders, ringworm, mouthwash [157], anti-inflammatory, respiratory disorders, dropsy, heart diseases, chronic fever, colic, scorpion stings, difficult urination, worm infestation [158], alopecia areata, erectile failure, boost appetite, abdominal pain, distaste, deworming, colitis [159], antitumor [160–163], ascites, edema [164]
30	*S. jabrense*		Brazil [165–169]	Anticancer [168], molluscicidal [169]
31	S. khasianum		India	Anti-inflammatory, antihelmintic, Anticancer [170–172]
32	S. laciniatum	Kangaroo apple	Australia, Tasmania, Wales, New Zealand [173, 174]	Unknown
33	*S. laxum*	Potato vine, potato climber, jasmine nightshade,	Australia [175, 176], Uruguay, Argentina [177, 178]	Aphid repellant pesticide [177]
34	*S. ligustrinum*	Natri, Tomatillo [541, 542]	Chile	Antipyretic, anti-inflammatory, fever, anti-fungal [179]
35	*S. lycocarpum*	Wolf apple, lobeira, fruit-of-wolf, jurubebao (Brazil) fruta-do-lobbo (Portuguese) [543]	Brazil	Anti-inflammatory, antihepatotoxic, hypotensive, antihistamine [180], anticancer [181], antidiabetic [182], antischistosomicidal [183, 184], antileishmanicidal [185], anti-trypanosomal [186] antiprotozoa [187]
36	*S. lycopersicum*	Tomatillo (Mexico), tomate (Spanish), tomato (English)	Mexico, South & Central America, Asia, Africa [188]	Antimicrobial [189], antiasthma, antiatherosclerosis [190], antiplatelet [191], anticancer [190, 192]
37	*S. lyratum*	Nipplefruit (English),	China South America [193]	Anticancer [88, 89, 194–200], anti-inflammatory [201]
38	*S. melongena*	Aubergine, bringal, eggplant, terong, baigan, melongene	India, China, Thailand, Burma, Iran, Egypt, Turkey, East Asia [202, 203]	Antioxidant [90, 91, 204–206], anticancer [206–208], antidiabetic [209], anti-inflammatory, analgesic, sedative, hypnotic, blood circulation [210], antimelanogenesis [211]
39	*S. muricatum*	Melon pear, Pepino, Tree melon, sweet cucumber [544–547]	Equador, Colombia, Peru, Chile, Sri Lanka, New Zealand, Western Australia, Spain, Israel, Morocco, Kenya, Hawaii, California [212, 213]	Anti-inflammatory [214], antidiabetic [215], antitumor [212, 213]
40	*S. nienkui*		China (Hainan) [216–218]	Unknown
41	*S. nigrum*	Black nightshade, duscle, garden nightshade, Indian nightshade, garden huckleberry, hound’s berry, petty morel, wonder berry, small-fruited black nightshade, or popolo, makoi (Hindi), manathakkali (Tamil)	Eurasia, Americas, Austrasia, South Africa [219–221]	Mouth ulcers, peptic ulcers, dysentery, skin disorders, ringworms, painful periods, cough [219–221], anti-inflammatory, hepatoprotective, diuretic, antipyretic, tuberculosis, cervical carcinoma [220–222], emollient, febrifuge, narcotic, purgative, sedative, analgesic, antispasmodic, vasodilator [222], antihyperlipidemic [131, 223], antimicrobial [224–226], antitumor [92–97, 227–230], anti-molluscicidal [231–233], antinociceptive, antipyretic [230, 234, 235], antiulcerogenic [235], antihistaminic, antiallergic [236, 237], hepatoprotective, anti-inflammatory, antipyretic [98, 236, 237], CNS-depressant action [238]
42	*S. nudum*		Caribbean, Haiti, Cuba [239]	Antiplasmodial [240–249]
43	*S. orbignianum*		Brazil [250]	Unknown
44	*S. paludosum*		Brazil	Hypertension, vasorelaxant, antioxidant, antibiotics [251, 252]
45	*S. paniculatum*	Jurubeba, jubeba, juribeba, juripeba, jupela, juripeba, juuna, juvena, jurubebinha, jurubeba-branca, jurubeba-verdadeira	Brazil, Argentina, Paraguay, southern, central, eastern and northern Brazil [253–255]	Anemia, anorexia, bile insufficiency, bladder problems, blood cleansing, bloating, boils, catarrh, congestion, contusions, constipation, convalescence, cystitis, debility, diabetes, digestive sluggishness, dyspepsia, edema, erysipelas, fever, flatulence, gallbladder inflammation, gastric disorders, hangover, headache, heartburn, hepatitis, hives, irritable bowel syndrome, itch, jaundice, liver problems, malaria, menstrual disorders, nausea, skin disorders, spleen inflammation, tumors, ulcers, water retention, wounds [253–255], antiherpes [256], antiulcers [257, 258], antifungal [259, 548], antibacterial [260]
46	*S. pseudocapsicum*	Jerusalem/winter cherry, Madeira,	South Africa, Australia, New Zealand, Peru, Ecuador [261–263]	Hepatoprotective [264]
47	*S. rostratum*	Buffalobur/spiny nightshade, Colorado bur, Kansas/Mexican/Texas thistle	United States, northern and central Mexico [265–272]	Cardiovascular [273]
48	*S. sarrachoides*	Hairy/leafy-fruited nightshade	Columbia [274, 275]	Unknown
49	*S. schimperianum*		Somali, Eritrea, Ethiopia, Egypt, Yemen [276]	Antimicrobial [277, 278], antifungal [279]
50	*S. septemlobum*	Qing qi (Chinese)	China (Anhui, Gansu, Hebei, Henan, Jiangsu, Liaoning, Nei Mongol, Shandong, Shanxi, Sichuan, Xinjiang, East Xizang, Zhejiang) [280, 281]	Antipyretic, antidotal [261], anticancer [261, 262]
51	*S. sessiliflorum*	Cocona	Peru, Colombia, Venezuela [282–284, 549], Bolivia, Mexico [268]	Antioxidant [550], antimicrobial, hypolipidemic [285]
52	*S. sisymbriifolium*	Vila-vila, sticky nighthade, red bufallor bur, fire and ice, litchi tomato, morelle de balbis	Brazil, Argentina, Uruguay, Paraguay [286–288]	Cardiovascular [289], antidiarrheal [290], hypotensive [291, 292], antimicrobial, antioxidative [293], anticonvulsant, CNS depressant [294], antimolluscicidal [295], analgesic [290, 296]
53	*S. spirale*		Southern China, India, Bangladesh, Thailand, Laos, Philippines, Australia [551]	Anaesthetic, diuretic and narcotic, antibacterial, anticancer [297–299]
54	*S. surattense*	Cockroach/yellow berry; thorn gourd/eggplant; belladonna; Night-shade, Febrifuge plant (English); Choti kateri/Bhatakataiyya, Rengani (Hindi);	China [300, 301], India [302]	Anti-inflammatory, antibacterial, antitumor, antioxidant, anti-platelet aggregation [303–308], diuretic [308], antiplasmodial [309], anthelmintic, anti-convulsant, antihyperlipide-mic, antiurolithiatic, natriuretic, antiulcer, wound healing, antiasthmatic, hypoglycemic, hepatoprotective [99]
55	*S. torvum*	Turkey berry, prickly nightshade, devil’s fig, shoo-shoo bush, wild/pea eggplant (English), aubergine sauvage épineuse, fausse aubergine (French), kantɔsi (Ghana), susumber (Jamaica), berenjena cimarrona (Spanish), kaisurisuri, kausoni, kauvotovotua, soni (Fijian), shui qie (Chinese), bhankatiya, katai (Hindi) [552]	Brazil, Colombia, Caribbean, Central America, Mexico, tropical Africa, Asia, Australia, Hawaii, Guam, American Samoa [310–312]	Antibacterial, anti-platelet aggregation [100, 313], pesticide [314], analgestic [314], anticancer [315–317], antifungal, antimicrobial [318–320], antiulcerogenic [321], antiviral [322], anticonvulsant [323], antihypertensive [324, 325, 553], antinephrotoxicity [326, 327], antioxidants [328–330], anti-inflammatory [331], antidepressant [332, 333], antiplasmodial [334], antidiabetic [335–337], antihelminthic [338]
56	*S. tridynamum*	Spanish: mala mujer, sacamanteca, ojo de liebre, berenjena Silvestre	Mexico [339, 340]	Antidiabetic [339–341]
57	*S. trilobatum*	Purple fruited pea eggplant, Thai nightshade	India, Myanmar, Thailand, Vietnam, Malaysia [342, 343]	Antifungal, antimitotic, asthma,vomiting, rheumatism, leprosy [342, 343], fever, antioxidant [344], antibacterial [345–347], antidiabetic [348], anticancer [349–355], mosquitocidal [356, 357], anti-inflamatory [358], antinociceptive [359], antihepatitis [360]
58	*S. triste*		Venezuela, Trinidad, Martinique, Dominica [361]	Unknown
59	*S. tuberosum*	Potato	Chile, Peru, Bolivia [101, 362, 363]	Antifungal, antimicrobial [364], antioxidants [365, 366], antileishmanial [367, 368], anticancer [369–372], antihypertensive [373]
60	*S. umbelliferum*	Bluewitch nightshade	California, Arizona [374–379]	Anticancer [380]
61	*S. uporo*	Cannibal’s tomato	Fiji island, Tonga, Samoa, Tuamotus, Hawaii [381–384]	Unknown
62	*S. validinervium*		Venezuela [385]	
63	*S. vestissimum*	Toronjo, tumo/coquina melon, lulo fruit	Colombia, Venezuela [386, 387]	
64	*S. villosum*	Hairy nightshade, whooly nightshade, red nightshade	Europe, western Asia, northern Africa, North America, Australia, India	Antimolluscicidal [554], mosquito larvicidal [388, 389, 555]
65	*S. violaceum*	Ci tian qie (Chinese)	China, India, Myanmar, Thailand, Cambodia, Laos, Vietnam, Malaysia, Indonesia, Philippines	Anticancer, anti-inflammatory, antimicrobial, antioxidant, anthelmintic [390–393]
66	*S. xanthocarpum*	Wild eggplant, Kantakari, yellow berried nightshade, huang shui qi (Chinese)	Nepal, Pakistan, Bhutan, Bangladesh, Myanmar, Sri Lanka, China, Iran, Yemen, Thailand, Afghanistan, Saudi Arabia, India	Anthelmintic, anti-inflammatory, anodyne, digestive, carminative, appetizer, stomachic, depurative, sudorific, febrifuge, expectorant, laxative, diuretic, emmenagogue, aphrodisiac, leishmaniasis, immunomodulatory, anti-asthmatic [394–400], antimicrobial [226, 401–405], molluscicidal, hepatoprotective, antidiabetic [406–413] antioxidant, antinociceptive, nephroprotective, mosquitocidal, anti-psoriatic, diuretic, antiurolithiatic [414–429]

## Chemical Constituents and Their Biological Properties

At least 670 compounds, including 134 steroidal saponins (**1**–**134**), 63 steroidal alkaloids (**135**–**197**), 13 pregnane glycosides (**198**–**210**), 128 terpenes (**211**–**338**), 72 flavonoids (**339**–**413**), 31 lignans (**414**–**444**), 31 other types of alkaloids (**445**–**475**), 66 sterols (**476**–**541**), 52 phenols (**542**–**593**), 20 coumarins and coumestans (**594**–**613**), 4 coumarinolignoids (**614**–**617**), 23 fatty acids and esters (**618**–**640**) and 30 other compounds (**641**–**670**) were reported from the genus *Solanum*. Most of them were investigated for various biological activities. The chemical constituents and their biological properties are presented in Table 2, together with their plant sources and parts, alongside the classification of structures.

**Table 2:** Phytochemistry, biological properties and classification of *Solanum* compounds

No.	Compounds	Plant sources	Parts	Biological properties	References
	*Steroidal Saponins*				
1	Chlorogenone	*S. torvum*	Fruit		[430]
2	(5*α*,25*S*)-Spirostan-3,6-dione	*S. torvum*	Fruit		[430]
3	Solakhasoside	*S. khasianum*	Fruit		[431]
4	Foliumin	*S. amygdalifolium*	Aerial		[57]
5	Foliumin A	*S. amygdalifolium*	Aerial		[56]
6	Neotigogenin	*S. paniculatum*	Leaf	Cytotoxic	[257]
7	Diuranthoside A	*S. cathayanum*	Root		[432]
8	Torvoside N	*S. torvum*	Aerial	Anticancer	[316]
9	Atroposide E	*S. dulcamara*	Aerial		[433]
10	Degalactotigonin	*S. dulcamara*	Aerial		[433]
11	Trillin	*S. paniculatum*	Aerial		[258]
12	Diosgenin gentiobioside	*S. paniculatum*	Aerial		[258]
13	Diosgenone	*S. nudum*	Leaf	Hepatoprotective	[242, 247, 249]
14	(22*R*, 23*S*, 25*R*)-3*β*,6*α*, 23-trihydroxy-5*α*-spirostane 6-*O*-*β*-d-xylosyl-(1*″”*-3*″’*)-*O*-[*β*-d-quinovosyl(1*″’*-2*′*)]-*O*-[*α*-l-rhamnosyl (1*″*-3*′*)] -*O*-*β*-d-quinovoside	*S. paniculatum*	Aerial		[258]
15	Nuatigenosido	*S. sisymbriifolium*	Root	Antihypertensive	[289, 291]
16	(3*β*,5*α*,14*β*,25*R*)-3-Hydroxyspirost-8-en-11-one	*S. villosum*	Leaf		[434]
17	(3*β*,5*α*,6*α*,25*S*)-3-Hydroxyspirostan-6-yl 6-deoxy-3-*O*-(6-deoxy-*α*-l-mannosyl) -*β*-d-glucoside	*S. torvum*	Whole		[435]
18	Torvoside Q	*S. torvum*	Aerial		[331, 436]
19	Dioscin	*S. indicum*	Fruit		[160]
		*S. melongena*	Fruit	Antimelanogenesis	[211]
		*S. rostratum*	Aerial		[437]
20	Prosapogenin A	*S. indicum*	Fruit		[160]
21	Diosgenin	*S. lycopersicum*	Aerial		[438]
		*S. melongena*	Aerial		[439]
		*S. nigrum*	Fruit		[440]
		*S. torvum*	Fruit		[430]
		*S. tridynamum*	Root		[341]
		*S. tuberosum*	Stem		[441]
		*S. violaceum*	Aerial		[391, 442]
22	Aspidistrin	*S. cathayanum*	Root		[432]
23	Torvoside M	*S. torvum*	Aerial	Anticancer	[316]
24	Protodioscin	*S. abutiloides*	Root		[7]
		*S. incanum*	Root		[156]
		*S. indicum*	Fruit		[160, 443]
		*S. spirale*	Fruit		[444]
25	Methylprotodioscin	*S. incanum*	Root		[155]
		*S. indicum*	Fruit		[160]
26	Indioside D	*S. incanum*	Root		[156]
27	26-*O*-*β*-d-Glucosyl-22-methoxyfurost-5-ene-3*β*,26-diol 3-*O*-*α*-l-rhamnosyl-(1-2)-*β*-d-glucoside	*S. indicum*	Fruit		[160]
		*S. spirale*	Fruit		[444]
28	(3*β*,22*α*,25*R*)-26-(*β*-d-Glucosyloxy)-22-hydroxyfurost-5-en-3-yl *O*-*β*-d-glucosyl-(1-2)-*O*-*β*-d-glucosyl-(1-4)–*β*-d-glucoside	*S. cathayanum*	Root		[432]
29	25*R*-Timosaponin H1	*S. cathayanum*	Root		[432]
30	Torvoside O	*S. torvum*	Leaf		[445]
31	(23*S*,25*R*)-spirost-5-en-3,23 diol 3-*O*-*α*-l-rhamnosyl-(1-2)-*O*-*α*-l-rhamnosyl-1-4)*β*-d-glucoside	*S. glabratum*	Aerial		[141]
32	23-*β*-d-glucosyl (23*S*,25*R*)spirost-5-en-3,23 diol 3-*O*-*α*-l-rhamnosyl-1-2)*O*-*α*-l-rhamnosyl-(1-4)*β*-d-glucoside	*S. glabratum*	Aerial		[141]
33	(25*R*)spirost-5-en-3-ol 3-*O*-*α*-l-rhamnosyl-1-2)*O*-*β*-d-glucosyl-1-3)*β*-d-galactoside	*S. glabratum*	Aerial		[141]
34	Isonuatigenin-3-*O*-*β*-solatriose	*S. sisymbriifolium*	Root		[446]
35	Saponin SC-1	*S. chrysotrichum*	Leaf		[118]
36	Saponin SC-2	*S. chrysotrichum*	Leaf	Antifungal	[113–115, 117]
37	Saponin SC-3	*S. chrysotrichum*	Leaf	Antifungal	[114, 117]
38	Saponin SC-4	*S. chrysotrichum*	Leaf	Antifungal	[114, 117]
39	Saponin SC-5	*S. chrysotrichum*	Leaf	Antifungal	[114, 117]
40	Saponin SC-6	*S. chrysotrichum*	Leaf	Antifungal	[114, 117]
		*S. torvum*	Whole		[435]
41	Chlorogenin	*S. chrysotrichum*	Leaf		[117]
		*S. tridynamum*	Root		[341]
		*S. torvum*	Fruit		[430]
42	Chrysogenin	*S. chrysotrichum*	Leaf		[117]
43	Laxumin A	*S. laxum*	Aerial		[178]
44	Laxumin B	*S. laxum*	Aerial		[178]
45	Luciamin	*S. laxum*	Aerial		[177]
46	Lyconoside Ia	*S. lycocarpum*	Fruit		[447]
47	Lyconoside Ib	*S. lycocarpum*	Fruit		[447]
48	Lyconoside II	*S. lycocarpum*	Fruit		[447]
49	Lyconoside III	*S. lycocarpum*	Fruit		[447]
50	Lyconoside IV	*S. lycocarpum*	Fruit		[447]
51	26-*O*-(*β*-d-Glucosyl) nuatigenin-3-*O*-*α*-l-rhamnosyl-(1-4)-*β*-d-glucoside	*S. surattense*	Aerial		[305]
52	Aculeatiside A	*S. surattense*	Aerial		[305]
53	(22R, 23S, 25R)-3*β*,6α,23-trihydroxy-5*α*-spirostane 6-*O*-*β*-d-xylosyl-(1-3) -*β*-d-quinovoside	*S. surattense*	Aerial		[305]
54	(22R,23S,25S)-3β,6α,23-trihydroxy-5α-spirostane 6-*O*-β-d-xylosyl-(1-3)-*O*-β-d-quinovoside	*S. surattense*	Aerial		[305]
55	(22R,23R,25S)-3β,6α,23-trihydroxy-5α-spirostane 6-*O*-β-d-xylosyl-(1-3)-*O*-β-d-quinovoside	*S. surattense*	Aerial		[305]
56	Neochlorogenin 6-*O*-*β*-d-quinovoside	*S. torvum*	Aerial		[331, 448]
57	Neochlorogenin 6-*O*-*β*-d-xylosyl -(1-3)-*β*-d-quinovoside	*S. torvum*	Aerial	Anti-inflammatory	[331, 448]
58	Neochlorogenin 6-*O*-*α*-l-rhamnosyl-(1-3)-*β*-d-quinovoside	*S. torvum*	Aerial		[448, 449]
59	Solagenin 6-*O*-*β*-d-quinovoside	*S. torvum*	Whole		[448–450]
60	Solagenin 6-*O*-*α*-l-rhamnosyl-(1-3)-*β*-d-quinovoside	*S. torvum*	Whole		[448]
61	(25*S*)26-*β*-d-glucosyloxy)3-oxo-5*α*-furost-20(22)en-6*α*-yl-*O*-*β*-d-xyloside	*S. torvum*	Fruit		[451]
62	(25*S*)26-*β*-d-glucosyloxy)3-oxo-22*α*-methoxy-5α-furostan-6*α*-yl-*O*-*β*-d-xyloside	*S. torvum*	Fruit		[451]
63	(25*S*)26-*β*-d-glucosyloxy)3*β*-hydroxy-22*α*-methoxy-5*α*-furostan-6*α*-yl-*O*-*α*-l-rhamnosyl-1-3)*β*-d-glucoside	*S. torvum*	Fruit		[451]
64	Torvoside A	*S. torvum*	Aerial		[313, 449]
65	Torvoside B	*S. torvum*	Root		[449]
66	Torvoside E	*S. torvum*	Root		[449]
67	Torvoside F	*S. torvum*	Root		[449]
68	Torvoside H	*S. torvum*	Fruit		[313]
69	(25*S*)3*β*-hydroxy-5α-spirostan-6*α*-yl-*O*-*β*-d-xyloside	*S. torvum*	Fruit		[451]
70	(25*S*)3-oxo-5α-spirostan-6*α*-yl-*O*-*β*-d-xyloside	*S. torvum*	Fruit		[451]
71	(25*S*)3*β*-hydroxy-5α-spirostan-6*α*-yl-*O*-*β*-d-glucoside	*S. torvum*	Fruit		[451]
72	(25*S*)3*β*,27-dihydroxy-5*α*-spirostan-6*α*-yl-*O*-*β*-d-glucoside.	*S. torvum*	Fruit		[451]
73	Neochlorogenin	*S. tridynamum*	Root		[451]
		*S. torvum*	Aerial		[341]
74	Tigogenin	*S. americanum*	Leaf		[54]
		*S. torvum*	Fruit		[430]
75	Yuccagenin	*S. tridynamum*	Root		[341]
76	Yamogenin	*S. violaceum*	Aerial		[391]
77	Yamogenone	*S. violaceum*	Aerial		[391]
78	Indioside L	*S. violaceum*	Aerial		[391]
79	Indioside M	*S. violaceum*	Aerial		[391]
80	Indioside N	*S. violaceum*	Aerial		[391]
81	Indioside O	*S. violaceum*	Aerial		[391]
82	Indioside G	*S. violaceum*	Whole		[392]
83	Indioside H	*S. violaceum*	Whole	Anticancer	[392]
84	Borassoside D	*S. violaceum*	Whole		[392]
85	Borassoside E	*S. violaceum*	Whole	Anticancer, anti-inflammatory	[392]
86	Indioside I	*S. violaceum*	Whole	Anticancer, anti-inflammatory	[392]
87	Indioside J	*S. violaceum*	Whole		[392]
88	Indioside K	*S. violaceum*	Whole		[392]
89	Yamoscin	*S. torvum*	Aerial	Anti-inflammatory	[331]
		*S. violaceum*	Whole	Anticancer	[392]
90	Zingiberoside A1	*S. violaceum*	Whole		[392]
91	Solanolactoside A	*S. torvum*	Aerial		[316]
92	Solanolactoside B	*S. torvum*	Aerial		[316]
93	Solanolactoside C	*S. torvum*	Aerial		[436]
94	Solanolide	*S. torvum*	Aerial		[316]
95	Torvoside J	*S. surattense*	Aerial	Anticonvulsant	[305]
		S. torvum	Aerial		[323, 331, 452]
96	Torvoside K	*S. surattense*	Aerial	Anticonvulsant, antifungal	[305]
		*S. torvum*	Aerial		[323, 331, 452]
97	Torvoside L	*S. surattense*	Aerial	Anticonvulsant	[305]
		*S. torvum*	Aerial		[323, 331, 435, 452]
		*S. paniculatum*	Leaf		[260]
98	(22R,23S,25S)-3β,6α,23-trihydroxy-5α-spirostane 6-*O*-β-d-xylosyl-(1-3)-*O*-β-d-quinovoside	*S. torvum*	Aerial		[323, 331]
99	(22R,23S,25R)-3β,6α,23-trihydroxy-5α-spirostane 6-*O*-β-d-xylosyl-(1-3)-*O*-β-d-quinovoside	*S. torvum*	Aerial	Anti-inflammatory	[331]
100	(22R,23R,25S)-3β,6α,23-trihydroxy-5α-spirostane 6-*O*-β-d-xylosyl-(1-3)-*O*-β-d-quinovoside	*S. torvum*	Aerial	Anti-inflammatory	[331]
101	Gekogenin	*S. torvum*	Fruit		[430]
102	Sisalagenin	*S. torvum*	Fruit		[430]
103	Δ^25(27)^tigogenin-3-*O*-*β*-d-glucoside	*S. paniculatum*	Leaf	Antiviral	[257]
104	Soladulcosides A	*S. dulcamara*	Aerial		[129]
105	Soladulcosides B	*S. dulcamara*	Aerial		[129]
106	Abutiloside L	*S. abutiloides*	Root		[4]
107	Abutiloside M	*S. abutiloides*	Root		[4]
108	Abutiloside N	*S. abutiloides*	Root		[4]
109	Abutiloside O	*S. abutiloides*	Root		[4]
110	Torvoside C	*S. torvum*	Root		[449]
111	Torvoside D	*S. surattense*	Aerial		[305]
		*S. torvum*	Root		[331, 449]
112	Torvoside G	*S. torvum*	Fruit, Root		[313, 449]
113	Torvoside P	*S. torvum*	Leaf		[445]
114	Anguivioside A	*S. anguivi*	Fruit		[41]
115	Anguivioside B	*S. anguivi*	Fruit		[41]
116	Anguivioside C	*S. anguivi*	Fruit		[41]
117	Anguivioside I	*S. indicum*	Fruit		[443]
118	Anguivioside III	*S. anguivi*	Fruit		[43]
		*S. indicum*	Fruit		[443]
119	Anguivioside XI	*S. anguivi*	Fruit		[43]
120	Anguivioside XV	*S. anguivi*	Fruit		[43]
121	Anguivioside XVI	*S. anguivi*	Fruit		[43]
122	Inunigroside A	*S. nigrum*	Fruit		[453]
123	25(*S*)-26-*O*-*β*-d-glucosyl-5*α*-furost-22(20)-en-3*β*,6α,26-triol 6-*O*-[*α*-l-rhamnosyl-(1-3)-*O*-*β*-d-quinovoside]	*S. torvum*	Fruit	Anticancer	[317]
124	25(*S*)-26-*O*-*β*-d-glucosyl-5*α*-furost-22(20)-en-3-one-6*α*,26-diol 6-*O*-[*α*-l-rhamnosyl-(1-3)-*O*-*β*-d-quinovoside]	*S. torvum*	Fruit	Anticancer	[317]
125	25(*S*)-26-*O*-*β*-d-glucosyl-5*α*-furost-22(20)-en-3*β*,6*α*,26-triol 6-*O*-*β*-d-quinovoside	*S. torvum*	Fruit	Anticancer	[317]
126	Paniculonin B	*S. torvum*	Leaf		[323]
127	Smilaxchinoside A	*S. rostratum*	Aerial		[437]
128	6-*O*-α-l-rhamnosyl-(1″-3′)-β-d-quinovosyl-(22*S*,23*R*,25*S*)-3*β*,6*α*,23-trihydroxy-5α-spirostane	*S. paniculatum*	Leaf		[260]
129	6-*O*-*β*-d-Xylosyl-(1″-3′)-*β*-d-quinovosyl-(23*R*,25*S*)-3*β*,6*α*,23-trihydroxy-5*α*-spirostane	*S. paniculatum*	Leaf		[260]
130	6-*O*-*β*-d-Xylosyl-(1″-3′)-*β*-d-quinovosyl-(22*S*,23*R*,25*R*)-3*β*,6*α*,23-trihydroxy-5*α*-spirostane	*S. paniculatum*	Leaf		[260]
131	3-*O*-*α*-l-Rhamnosyl-(1″-3′)-*β*-d-quinovosyl-(22*S*,23*S*,25*R*)-3*β*,6*α*,23-trihydroxy-5*α*-spirostane	*S. paniculatum*	Leaf		[260]
132	3-*O*-*β*-d-Xylosyl-(1″-3′)-*β*-d-quinovosyl-(22*S*,23*S*,25*R*)-3*β*,6*α*,23-trihydroxy-5*α*-spirostane	*S. paniculatum*	Leaf		[260]
133	6-*O*-*α*-l-Rhamnosyl-(1″-3′)-*β*-d-quinovosyl-(22*S*,25*S*)-1*β*,3*β*,6*α*-trihydroxy-5α-spirostane	*S. paniculatum*	Leaf		[260]
134	6-*O*-*β*-d-Xylosyl-(1″-3′)-*β*-d-quinovosyl-(22S,25*S*)-3*β*,4*β*,6*α*-trihydroxy-5*α*-spirostane	*S. paniculatum*	Leaf		[260]
	*Steroidal alkaloids*				
135	Demissine	*S. tuberosum*	Stem		[101]
136	Solasodiene	*S. torvum*	Fruit		[430]
137	Solanoside A	*S. surattense*	Whole		[454]
138	Solanoside B	*S. surattense*	Whole		[454]
139	Solamargine	*S. abutiloides*	Root		[7]
		*S. aculeastrum*	Fruit		[19]
		*S. asperum*	Root		[66, 67]
		*S. buddleifolium*	Stem		[79]
		*S. americanum*	Fruit		[55]
		*S. anguivi*	Root		[42]
		*S. crinitum*	Fruit		[122]
		*S. erianthum*	Leaf		[137, 455]
		*S. incanum*	Root		[156]
		*S. khasianum*	Fruit		[456]
		*S. lycocarpum*	Fruit	Leishmanicidal, antidiabetic, schistosomicidal, trypanocidal	[182, 183, 185, 186, 447, 457]
		*S. melongena*	Fruit,Root		[206, 439]
		*S. nigrum*	Whole		[228]
		*S. paludosum*	Fruit		[253]
		*S. sarrachoides*	Leaf	Anticancer	[458]
		*S. surattense*	Aerial		[305]
		*S. uporo*	Root	Antibacterial, molluscicidal	[384]
		*S. xanthocarpum*	Fruit		[403, 406]
140	*γ*-Solamargine	*S. nigrum*	Whole		[228]
		*S. umbelliferum*	Whole		[380]
141	Khasianine	*S. khasianum*	Fruit		[456]
		*S. nigrum*	Whole		[228]
		*S. surattense*	Aerial	Anticancer	[305]
		*S. xanthocarpum*	Fruit	Antibacterial, molluscicidal	[403, 406, 407]
142	Solasonine	*S. americanum*	Leaf		[54]
		*S. amygdalifolium*	Aerial		[56]
		*S. asperum*	Fruit		[66, 67]
		*S. crinitum*	Aerial		[122, 123, 459]
		*S. erianthum*	Leaf		[137, 455]
		*S. khasianum*	Fruit		[456]
		*S. lycocarpum*	Fruit	Leishmanicidal,antidiabetic, schistosomicidal	[182, 183, 185, 447, 457]
		*S. melongena*	Fruit,Root		[206, 439]
		*S. sarrachoides*	Leaf		[458]
		*S. sessiliflorum*	Fruit		[460]
		*S. sisymbriifolium*	Fruit		[294]
143	*β*1-Solasonine	*S. nigrum*	Whole		[228]
144	12-Hydroxysolasonine	*S. lycocarpum*	Fruit		[182, 447]
145	Solasodine				
		*S. americanum*	Leaf		[54]
		*S. aculeastrum*	Fruit	Anticancer	[13]
		*S. crinitum*	Aerial		[123]
		*S. khasianum*	Fruit		[172, 456]
		*S. laciniatum*	Aerial		[461, 462]
		*S. lycocarpum*	Fruit		[185]
		*S. melongena*	Fruit		[206]
		*S. nigrum*	Whole		[163, 440]
		*S. sisymbriifolium*	Fruit		[294]
		*S. surattense*	Whole	CNS depressant	[303]
		*S. torvum*	Whole	Anti-inflammatory	[463]
		*S. trilobatum*	Whole		[358]
		*S. villosum*	Whole		[442]
		*S. xanthocarpum*	Fruit	Antibacterial	[403, 429]
		*S. umbelliferum*	Whole		[380]
146	*N*-Hydroxysolasodine	*S. paludosum*	Root		[464]
147	*O*-Acetylsolasodine	*S. umbelliferum*	Whole		[380]
148	Putuline	*S. paludosum*	Root		[464]
149	Anguivine	*S. anguivi*	Root		[42]
		*S. uporo*	Root		[384]
150	Isoanguivine	*S. uporo*	Root		[384]
151	Arudonine	*S. arundo*	Root		[64]
152	Solanandaine	*S. asperum*	Fruit		[66]
153	Robeneoside A	*S. lycocarpum*	Fruit		[182, 447]
154	Robeneoside B	*S. lycocarpum*	Fruit		[182, 447]
155	Lobofrutoside	*S. lycocarpum*	Fruit		[447]
156	Solanigroside P	*S. nigrum*	Whole		[228]
157	(22R, 25R)-16β-H-22α-*N*-Spirosol-3β-ol-5-ene 3-*O*-α-l-rhamnosyl-(1-2)-[α-l-rhamnosyl-(1-4)]-β-d-glucoside	*S. surattense*	Aerial	Anticancer	[305]
158	Solaculine A	*S. aculeastrum*	Root		[19]
159	*β*-Solamarine	*S. aculeastrum*	Root		[19]
		*S. elaeagnifolium*	Seed		[465]
		*S. incanum*	Root		[155]
160	Tomatidenol	*S. aculeastrum*	Root		[19]
		*S. palodusum*	Root		[464]
		*S. lycopersicum*	Fruit		[192]
		*S. surattense*	Aerial		[454]
161	Tomatidine 3-*O*-*β*-d-glucoside	*S. arboreum*	Aerial		[63]
162	Dehydrotomatine	*S. lycopersicum*	Fruit		[192]
163	Tomatidine 3-*O*–*O*-*β*-d-xylosyl-1-6)*β*-d-glucoside]	*S. arboreum*	Aerial		[63]
164	Solaverol A	*S. uporo*	Root		[384]
165	(23S)-23-hydroxyanguivine	*S. uporo*	Root		[384]
166	(23S)-23-hydroxyisoanguivine	*S. uporo*	Root		[384]
167	Tomatidine	*S. lycopersicum*	Fruit		[192]
		*S. aculeastrum*	Fruit	Anticancer	[13]
168	Tomatine	*S. lycopersicum*	Fruit		[192, 466]
		*S. cathayanum*	Whole	Neurotoxicity	[106]
		*S. sarrachoides*	Leaf		[276]
169	22-Imido-3-[4′-(6″-deoxy-*α*-l-mannoside)-*β*-d-glucoside]-5-dehydro spirostane	*S. xanthocarpum*	Fruit		[407]
170	Leptinidine	*S. paludosum*	Root		[253]
		*S. orbignianum*	Aerial		[250]
171	Leptinine I	*S. orbignianum*	Aerial		[250]
172	Leptinine II	*S. orbignianum*	Aerial		[250]
173	Solanine	*S. dulcamara*	Stem		[467]
		*S. indicum*	Whole		[162]
		*S. tuberosum*	Stem		[441]
		*S. villosum*	Fruit		[468]
174	*α*-Chaconine	*S. tuberosum*	Stem		[372, 441]
175	*β*-d-Glucoside, (3*β*,23*β*)23-hydroxysolanid-5-en-3-yl	*S. orbignianum*	Aerial		[250]
176	Solanidine	*S. villosum*	Fruit		[469]
177	Solanopubamine	*S. schimperianum*	Aerial	Antifungal	[279]
178	Jurubine	*S. paniculatum*	Fruit		[273, 548]
179	Etioline	*S. spirale*	Root		[470]
180	Deacetylveralosine	*S. spirale*	Root		[470]
		*S. diphyllum*	Root		[126]
181	Solaspiralidine	*S. spirale*	Root		[470]
182	Soladunalinidine	*S. arboreum*	Aerial		[59]
183	3-*epi*-Soladunalinidine	*S. arboreum*	Aerial		[59]
184	Caavuranamide	*S. caavurana*	Fruit	Antibacterial	[80]
185	4-Tomatiden-3-one	*S. caavurana*	Fruit		[80]
186	5-Tomatidan-3-one	*S. caavurana*	Fruit		[80]
187	(22S,25S)-3β-aminospirosol-5-ene	*S. arboreum*	Aerial		[59]
188	(22*R*,25*R*)3*β*-amino-5α-spirosolane	*S. triste*	Aerial		[362, 471]
189	(22*R*,25*R*)3*β*-amino-5-spirosolene	*S. triste*	Aerial		[362, 471]
190	Isojuripidine	*S. asterophorum*	Aerial	Spasmolytic	[70]
191	23,24-2-methyl-tetrahydrofuran)Solanidine	*S. cornifolium*	Aerial		[472, 473]
192	Spiraloside C	*S. spirale*	Fruit		[474]
193	Spiraloside B	*S. spirale*	Fruit		[474]
194	Spiraloside A	*S. spirale*	Fruit		[474]
195	Soladulcine A	*S. dulcamara*	Aerial		[433]
196	Soladulcine B	*S. dulcamara*	Aerial		[433]
197	Esculeoside A	*S. lycopersicum*	Fruit		[475]
	*Pregnane glycosides*				
198	Solanigroside A	*S. nigrum*	Whole		[476]
199	Solanigroside B	*S. nigrum*	Whole		[476]
200	5*α*-Pregn-16-en-3*β* -ol-20-one lycotetraoside	*S. nigrum*	Whole		[476]
201	(5*α*)-3-Hydroxypregn-16-en-20-one	*S. lyratum*	Whole		[194]
202	Hypoglaucin H	*S. nigrum*	Whole		[476]
		*S. rostratum*	Aerial		[437]
203	16-Dehydropregnolone	*S. lyratum*	Whole	Anticancer	[194]
204	16-dehydropregnenolone 3-*O*-α-l-rhamnosyl-1-2)*β*-d-glucosiduronic acid	*S. lyratum*	Whole		[194]
205	Torvpregnanoside A	*S. torvum*	Aerial		[317, 331]
206	5*α*-pregn-16-en-3,20-dione-6*α*-ol-6-*O*-[*α*-l-rhamnosyl-(1-3)-*β*-d-quinovoside]	*S. torvum*	Fruit	Anticancer	[317]
207	Torvpregnanoside B	*S. torvum*	Aerial		[331]
208	Ganaxolone	*S. torvum*	Aerial		[323]
209	Allopregnanolone	*S. torvum*	Aerial		[323]
210	Pregnanolone	*S. torvum*	Aerial		[323]
	*Triterpenes*				
211	Betulinic acid	*S. buddleifolium*	Stem		[79]
212	Lupeol	*S. cathayanum*	Aerial		[472, 473, 477]
		*S. schimperianum*	Aerial		[278]
		*S. spirale*	Leaf	Anticancer	[297]
213	Cycloeucalenone	*S. cernuum*	Leaf	Anticancer	[107]
214	24-oxo-31-norcycloartanone	*S. cernuum*	Leaf	Anticancer	[107]
215	Friedelin	*S. lycopersicum*	Seed		[478]
216	Ursolic acid	*S. lyratum*	Whole		[197]
		*S. torvum*	Aerial		[463]
		*S. xanthocarpum*	Root		[427]
217	2*α*,3*β*-Dihydroxyursolic acid	*S. torvum*	Aerial		[463]
218	Daturaolone	*S. arundo*	Whole		[65]
219	Carbenoxolone	*S. cernuum*	Leaf		[109]
220	*β*-Amyrin	*S. melongena*	Aerial		[439]
221	Oleanolic acid	*S. torvum*	Aerial		[463]
		*S. xanthocarpum*	Root		[427]
222	2*α*-Hydroxyoleanolic acid	*S. torvum*	Aerial		[463]
223	3*β*-Acetoxy-11*α*,12*α*-epoxyoleanan-13ß,28-olide	*S. torvum*	Aerial		[463]
224	Solanoglycosydane I	*S. torvum*	Fruit		[314]
	*Diterpenes*				
225	Phytol	*S. pseudocapsicum*	Leaf		[263]
		*S. villosum*	Leaf		[434, 479]
226	Kaur-16-ene	*S. aculeastrum*	Leaf		[11]
227	Solanerioside A	*S. erianthum*	Leaf		[138]
228	Tricalysioside U	*S. violaceum*	Whole		[392]
	*Sesquiterpenes*				
229	Roseoside	*S. erianthum*	Leaf		[138]
230	(6*E*,10*E*)-5,12-Dihydroxy-ß-nerolidol 5-*O*-*β*-d-glucoside	*S. erianthum*	Leaf		[138]
231	Amarantholidoside IV	*S. erianthum*	Leaf		[138]
232	3*β*-Hydroxysolavetivone	*S. abutiloides*	Root	Antifungal	[3]
		*S. aethiopicum*	Root		[29]
233	Solavetivone	*S. abutiloides*	Root	Antifungal	[3]
		*S. aethiopicum*	Root		[29]
		*S. indicum*	Root		[163]
		*S. jabrense*	Aerial		[166]
234	13-Hydroxysolavetivone	*S. buddleifolium*	Stem		[79]
		*S. aethiopicum*	Root		[29]
235	Lubimin	*S. abutiloides*	Root	Antifungal	[3]
		*S. aethiopicum*	Root		[29]
236	Lubiminoic acid	*S. aethiopicum*	Root		[29]
237	Epilubimin	*S. aethiopicum*	Root		[29]
238	Epilubiminoic acid	*S. aethiopicum*	Root		[29]
239	Lubiminol	*S. aethiopicum*	Root		[29]
240	*α*-Farnesene	*S. aculeastrum*	Leaf		[11]
241	Nerolidol	*S. aculeastrum*	Leaf		[11]
242	2,7,10-Trimethyldodecane	*S. aculeastrum*	Leaf		[11]
243	Aethione	*S. aethiopicum*	Root		[29]
244	Anhydro-*β*-rotunol	*S. aethiopicum*	Root		[29]
245	(4*S*,5*R*,7*S*)-4,11-Dihydroxy-guaia-1(2),9(10)-dien	*S. erianthum*	Stem		[480]
246	Caryophyllene	*S. erianthum*	Fruit		[481]
247	Cadina-1(10),4-diene	*S. erianthum*	Fruit		[481]
248	*α*-Gurjunene	*S. erianthum*	Fruit		[481]
249	Globulol	*S. erianthum*	Fruit		[481]
250	α-Guaiene	*S. erianthum*	Fruit		[481]
251	α-Calacorene	*S. erianthum*	Fruit		[481]
252	2-naphthalenemethanol	*S. erianthum*	Fruit		[481]
253	Octahydro-2,2-dimethyl-4a,7a-ethano-5*H*-cyclobut[*e*]inden-5-ol	*S. erianthum*	Fruit		[481]
254	4,5-Dehydroisolongifolene	*S. erianthum*	Fruit		[481]
255	*α* -Caryophyllene	*S. erianthum*	Fruit		[481]
256	Solafuranone	*S. indicum*	Root		[163]
257	Lyratol D	*S. lyratum*	Whole	Anticancer	[199]
		*S. septemlobum*	Whole		[482]
258	Solajiangxin B	*S. lyratum*	Whole	Anticancer	[198]
		*S. septemlobum*	Whole		[482]
259	Septemlobin D	*S. septemlobum*	Whole		[483]
260	Blumenol A	*S. lyratum*	Whole	Anticancer	[199, 484]
261	Blumenol C	*S. lyratum*	Whole		[484]
262	Dehydrovomifoliol	*S. lyratum*	Whole	Anticancer	[199, 484]
263	Grasshopper ketone	*S. lyratum*	Whole		[484]
264	6*α*-Epoxy-7-megastigmen-9-one	*S. lyratum*	Whole		[484]
265	(1′*R*,2*R*,5*S*,10*R*)2-1′,2′-dihydroxy-1′-methylethyl)6,10-dimethylspiro[4, 5]dec-6-en-8-one	*S. lyratum*	Whole		[484]
266	(1′*S*,2*R*,5*S*,10*R*)2-1′,2′-dihydroxy-1′-methylethyl)6,10-dimethylspiro[4, 5]dec-6-en-8-one	*S. lyratum*	Whole		[484]
267	2-1′,2′-dihydroxy-1′-methylethyl)6,10-dimethyl-9-hydroxyspiro[4, 5]dec-6-en-8-one	*S. lyratum*	Whole		[200, 484]
268	Boscialin	*S. lyratum*	Whole		[484]
269	1*β*-Hydroxy-1,2-dihydro-*α*-santonin	*S. lyratum*	Whole		[193, 484]
270	Lyratol A	*S. lyratum*	Whole		[485]
271	Lyratol B	*S. lyratum*	Whole		[485]
		*S. septemlobum*	Whole		[482]
272	Lyratol C	*S. lyratum*	Whole	Anticancer	[199]
273	Lyratol G	*S. lyratum*	Whole		[196]
274	Solajiangxin A	*S. lyratum*	Whole	Anticancer	[198]
275	Solajiangxin C	*S. lyratum*	Whole	Anticancer	[198]
276	Solajiangxin D	*S. lyratum*	Whole	Anticancer	[200]
		*S. septemlobum*	Whole		[482]
277	Solajiangxin E	*S. lyratum*	Whole	Anticancer	[200]
278	Solajiangxin F	*S. lyratum*	Whole	Anticancer	[197]
		*S. septemlobum*	Whole		[482]
279	Solajiangxin G	*S. lyratum*	Whole	Anticancer	[197]
280	2-hydroxysolajiangxin E	*S. lyratum*	Whole	Anticancer	[200]
281	Dehydrocarissone	*S. lyratum*	Stem		[486]
		*S. septemlobum*	Whole		[482]
282	Atractylenolide I	*S. lyratum*	Stem		[486]
283	Ligucyperonol	*S. septemlobum*	Whole		[482]
284	Nardoeudesmol A	*S. septemlobum*	Whole		[482]
285	Solanerianone A	*S. septemlobum*	Whole		[482]
286	Pterocarptriol	*S. torvum*	Root		[487]
287	Selina-3*β*,4*α*,11-triol	*S. torvum*	Root		[487]
288	2-(1′,2′-dihydroxy-1′-methylethyl)-6,10-dimethylspiro[4, 5]dec-6,9-dien-8-one	*S. torvum*	Root		[487]
289	10*β*,12,14-Trihydroxy-*allo*-aromadendrane	*S. torvum*	Root		[487]
290	10*β*,13,14-Trihydroxy-*allo*-aromadendrane	*S. torvum*	Root		[487]
291	2-(1′,2′-dihydroxy-1′-methylethyl)-6,10-dimethyl-9-hydroxy-spirodec-6-en-8-one	*S. torvum*	Root		[487]
292	1*β*,10*β*,12,14-Tetrahydroxy-*allo*-aromadendrane	*S. torvum*	Root		[487]
293	1*β*,10*β*,13,14-Tetrahydroxy-*allo*-aromadendrane	*S. torvum*	Root		[487]
294	Teferidin	*S. schimperianum*	Aerial		[278]
295	Teferin	*S. schimperianum*	Aerial		[278]
296	Ferutinin	*S. schimperianum*	Aerial		[278]
297	Bisabolol	*S. sessiliflorum*	Fruit		[488]
298	11,12-*O*-Isopropylidenesolajiangxin F	*S. septemlobum*	Whole		[483]
299	Eudesmane	*S. septemlobum*	Whole		[281]
300	Vitispirane	*S. septemlobum*	Whole		[281]
301	Septemlobin A	*S. septemlobum*	Whole	Anticancer	[281]
302	Septemlobin B	*S. septemlobum*	Whole	Anticancer	[281]
303	Septemlobin C	*S. septemlobum*	Whole	Anticancer	[281]
304	3*β*,11-dihydroxy-4,14-oxideenantioeudesmane	*S. torvum*	Root		[487]
305	Aromadendrene oxide	*S. erianthum*	Fruit		[481]
306	Thujopsene	*S. betaceum*	Fruit		[77]
307	*α*-Cedrene	*S. betaceum*	Fruit		[77]
308	Cedrol	*S. betaceum*	Fruit		[77]
309	*α*-Hexylcinnamaldehyde	*S. betaceum*	Fruit		[77]
310	*β*-Cadinene	*S. betaceum*	Fruit		[77]
	*Monoterpenes*				
311	Decanal	*S. aculeastrum*	Leaf		[11]
312	Decane	*S. aculeastrum*	Leaf		[11]
313	2,4-Decadienal	*S. aculeastrum*	Leaf		[11]
314	1,8-Cineole	*S. betaceum*	Fruit		[77]
315	Terpinen-4-ol	*S. betaceum*	Fruit		[77]
316	Linalool	*S. vestissimum*	Fruit		[489, 490]
317	Geraniol	*S. vestissimum*	Fruit		[490]
318	Limonene	*S. vestissimum*	Fruit		[490]
319	*β*-Cyclocitral	*S. aculeastrum*	Leaf		[11]
320	*β*-Ionone	*S. aculeastrum*	Leaf		[11]
		*S. pseudocapsicum*	Leaf		[263]
		*S. betaceum*	Fruit		[77]
321	1, 2-Dihydro-1,1,6-trimethyl-naphthalene	*S. aculeastrum*	Leaf		[11]
322	*trans*-*β* -Damascenone	*S. aculeastrum*	Leaf		[11]
323	Loliolide	*S. erianthum*	Leaf		[137]
		*S. americanum*	Aerial		[49]
		*S. pseudocapsicum*	Leaf		[263]
324	Hotrienol	*S. vestissimum*	Fruit		[468, 490]
325	Neroloxide	*S. vestissimum*	Fruit		[468]
326	5-Ethynyltetrahydro-α,α,5-trimethyl-2-furanmethanol	*S. vestissimum*	Fruit		[490]
327	Nerol	*S. vestissimum*	Fruit		[490]
328	8-Hydroxylinalool	*S. vestissimum*	Fruit		[491]
329	(*R*)-Linalyl *β*-d-glucoside	*S. vestissimum*	Fruit		[492]
330	(1*R*,4*E*)-1-Ethenyl-6-hydroxy-1,5-dimethyl-4-hexen-1-yl *β*-d-glucoside	*S. vestissimum*	Fruit		[492]
331	(*R*)-Linalyl *β*-vicianoside	*S. vestissimum*	Fruit		[492]
332	6-*O*-linked *β*-d-glucoside of (*R*)*E*)2,6-dimethyl-3,7-octadiene-2,6-diol	*S. vestissimum*	Fruit		[468]
333	(3*E*,6*R*)-2,6-Dimethyl-3,7-octadiene-2,6-diol	*S. vestissimum*	Fruit		[468]
334	*p*-Cymenene	*S. betaceum*	Fruit		[77]
335	Dihydroactinidiolide	*S. erianthum*	Leaf		[137]
336	Apiole	*S. sessiliflorum*	Fruit		[488]
337	*α*-Terpinen-7-al	*S. betaceum*	Fruit		[77]
338	1,3,8-*p*-Menthatriene	*S. betaceum*	Fruit		[77]
	*Flavonoids*				
339	Vitecetin	*S. agrarium*	Aerial		[31]
340	Quercetin	*S. anguvi*	Fruit	Anticancer	[31]
		*S. elaeagnifolium*	Seed		[493]
		*S. incanum*	Aerial		[494]
		*S. melongena*	Stem		[205]
		*S. muricatum*	Whole		[215]
		*S. nigrum*	Leaf		[92–98, 230–238, 495–497]
		*S. torvum*	Whole		[498]
341	Kaempferol 7-*O*-rhamnoside	*S. asperum*	Fruit		[67]
342	Rutin	*S. anguvi*	Fruit	Anticancer	[31]
		*S. melongena*	Stem		[499, 500]
		*S. muricatum*	Fruit		[215]
		*S. nigrum*	Leaf		[230]
		*S. spirales*	Aerial		[470]
343	Kaempferol 3-rutinoside-7-rhamnoside	*S. asperum*	Fruit		[67]
344	Afzelin	*S. cernuum*	Leaf		[109, 112, 501]
345	Quercitrin	*S. cernuum*	Leaf		[109]
		*S. melongena*	Stem		[205]
346	Astragalin	*S. cernuum*	Leaf		[501]
		*S. crinitum*	Aerial		[459]
		*S. incanum*	Aerial		[494]
		*S. elaeagnifolium*	Aerial		[502]
347	Kaempferol 3-*O*-[*α*-apiofuranosyl-(1-2)]-*α*-rhamnoside	*S. cernuum*	Leaf		[501]
348	Kaempferol 3-*O*-[*α*-apiofuranosyl-(1-2)]-*β*-galactoside	*S. cernuum*	Leaf		[501]
349	Tiliroside	*S. asperum*	Fruit		[67]
		*S. crinitum*	Aerial		[123, 459]
		*S. elaeagnifolium*	Whole	Anticancer	[503]
		*S. cernuum*	Leaf		[501]
350	*cis*-Tiliroside	*S. cernuum*	Leaf		[501]
		*S. elaeagnifolium*	Aerial		[502]
351	Kaempferol	*S. crinitum*	Aerial		[459]
		*S. elaeagnifolium*	Whole		[504]
		*S. incanum*	Aerial		[494]
		*S. indicum*	Whole		[505]
		*S. nigrum*	Leaf		[227]
		*S. surattense*	Whole		[99]
		*S. torvum*	Whole		[498]
352	Camelliaside C	*S. erianthum*	Leaf		[137]
353	Baimaside	*S. incanum*	Aerial		[506]
354	Narcissin	*S. glabratum*	Aerial		[141]
355	Isorhamnetin 3-glucoside	*S. incanum*	Aerial		[506]
356	Populnin	*S. elaeagnifolium*	Aerial		[502]
357	Quercetin 3-*O*-robinoside	*S. paniculatum*	Aerial		[258]
358	Kaempferol 3-*O*-(6″-*O*-cis-*p*-coumaroyl)-*O*-*β*-galactoside	*S. elaeagnifolium*	Aerial		[502]
359	Myricetin-3-galactoside	*S. melongena*	Stem		[205]
360	Apigenin	*S. lyratum*	Whole		[507]
		*S. torvum*	Whole		[498]
361	Pelanin	*S. tuberosum*	Stem		[508]
362	Petanin	*S. tuberosum*	Stem		[508]
363	Peonanin	*S. tuberosum*	Stem		[508]
364	Keracyanin	*S. betaceum*	Fruit	Anticancer	[75, 76]
365	Pelargonidin 3-rutinoside	*S. betaceum*	Fruit	Anticancer	[75, 76]
366	Tulipanin	*S. betaceum*	Fruit	Anticancer	[75, 76]
367	Delphinidin 3-*O*-α-l-rhamnosyl-(1-6)-*β*-d-glucoside-3′-*O*-*β*-d-glucoside	*S. betaceum*	Fruit	Anticancer	[75, 76]
368	Cyanidin 3-*O*-(2″-*O*-xylosyl)rutinoside	*S. betaceum*	Fruit		[76]
369	Asterin	*S. betaceum*	Fruit		[76]
370	Biochanin A-7-*O*-*β*-d-apiofuranosyl-1-5)*β*-d-apiofuranosyl-1-6)*β*-d-glucoside	*S. crinitum*	Fruit		[122]
371	2R,3R-5,7,4′-trihydroxy-dihydroflavon-3-*O*-α-d-glucosyl-6″-*O*-β-d-glucoside-6‴-p-hydroxy benzoate	*S. elaeagnifolium*	Whole	Anticancer	[503]
372	6,2′,3″,5″,4‴-Pentahydroxy-3,7″-biflavone	*S. dulcamara*	Fruit		[130]
373	Kaempferol 8-*C*-*β*-d-galactoside	*S. elaeagnifolium*	Aerial	Hepatoprotective	[502]
374	Kaempferol 8-*C*-glucoside	*S. elaeagnifolium*	Aerial		[502]
375	Kaempferol 6-*C*-glucoside	*S. elaeagnifolium*	Aerial		[502]
376	Vitexin	*S. elaeagnifolium*	Aerial		[502]
377	Vicenin II	*S. elaeagnifolium*	Aerial		[502]
378	Quercetin 6-*C*-*β*-glucoside	*S. elaeagnifolium*	Aerial		[502]
379	Quercetin 3-*O*-*β*-galactoside	*S. elaeagnifolium*	Aerial		[502]
380	Isoquercitrin	*S. elaeagnifolium*	Aerial		[502–504]
		*S. incanum*	Aerial		[494]
		*S. torvum*	Root		[338]
		*S. melongena*	Stem		[205]
381	Quercetin 3-*O*-*β*-apiofuranosyl-(1-2)-*O*-*β*-galactoside	*S. elaeagnifolium*	Aerial		[502]
382	5-Hydroxy,7,2′,3′,5′-tetramethoxyflavone	*S. glabratum*	Whole		[140]
383	Combretol	*S. glabratum*	Whole		[140]
384	Baicalin	*S. incanum*	Aerial		[506]
385	Kaempferol 3‐*O*‐(6‴‐*O*‐2,5‐dihydroxycinnamoyl)‐*β*‐D‐glucosyl(1-2) *β*‐D‐glucoside	*S. incanum*	Aerial		[506]
386	(±)-Naringenin	*S. indicum*	Whole		[505]
		*S. nienkui*	Whole		[509]
		*S. sessiliflorum*	Fruit		[510]
		*S. surattense*	Whole		[99]
387	Manghaslin	*S. lycopersicum*	Fruit		[511]
388	Genkwanin	*S. jabrense*	Aerial		[167]
		*S. palodusum*	Aerial		[512]
389	Ombuine	*S. jabrense*	Aerial		[167]
390	Rhamnocitrin	*S. jabrense*	Aerial		[167]
		*S. palodusum*	Aerial		[513]
391	Retusin	*S. jabrense*	Aerial		[167]
		*S. palodusum*	Aerial		[512]
		*S. schimperianum*	Aerial		[278]
		*S. torvum*	Fruit		[322]
392	Pentamethoxyquercetin	*S. jabrense*	Aerial		[167]
393	3-*O*-Methylquercetin	*S. jabrense*	Aerial		[167]
		*S. palodusum*	Aerial		[513]
394	Kumatakenin	*S. jabrense*	Aerial		[167]
		*S. palodusum*	Aerial		[513]
395	3′-Hydroxyflindulatin	*S. jabrense*	Aerial		[167]
		*S. palodusum*	Aerial		[513]
396	3,7,8-Trimethylherbacetin	*S. jabrense*	Aerial		[167]
397	3,7,8,3′,4′-Pentamethylgossypetin	*S. jabrense*	Aerial		[167]
		*S. palodusum*	Aerial		[512, 513]
398	Diosmetin	*S. nienkui*	Whole		[509]
399	Formononetin	*S. lyratum*	Whole		[514]
400	Ononin	*S. lyratum*	Whole		[514]
401	Daidzein	*S. lyratum*	Whole		[507, 514]
402	Genistin	*S. lyratum*	Whole		[514]
403	5-Hydroxylononin	*S. lyratum*	Whole		[514]
404	2,7-Dihydroxy-3-(4-hydroxyphenyl)-5-methoxy-4*H*-1-benzopyran-4-one	*S. nienkui*	Whole		[509]
405	5-hydroxy-3,7,4′-trimethoxyflavone	*S. schimperianum*	Aerial		[278]
406	Kaempferol-3-*O*-*β*-d-glucoside	*S. schimperianum*	Aerial		[278]
407	Luteolin	*S. schimperianum*	Aerial		[278]
408	Tamarixin	*S. torvum*	Whole		[498]
409	Torvanol A	*S. torvum*	Root	Antidepressant, antiviral	[322, 332]
410	5-methoxy-(3,4″-dihydro-3″,4″-diacetoxy)-2″,2′-dimethyl-(7,8:5″,6″)-flavone	*S. erianthum*	Leaf		[137]
411	5,7,8,4′-tetrahydroxy-3-methoxyflavone-8-*O*-β-d-xyloside	*S. rostratum*	Aerial		[515]
412	3-*O*-Methylquercetin 3-*O*-*β*-d-galactoside	*S. rostratum*	Whole		[516]
413	3-*O*-Methylquercetin 3-*O*-*β*-d-glucoside	*S. rostratum*	Whole		[516]
	*Lignans*				
414	Isolariciresinol	*S. buddleifolium*	Stem		[79]
415	5-Methoxyisolariciresinol	*S. buddleifolium*	Stem		[79]
416	Polystachyol	*S. buddleifolium*	Stem		[79]
417	(+)-Lyoniresinol 3-*O*-d-glucoside	*S. buddleifolium*	Stem		[79]
418	(-)-Lyoniresinol 3-*O*-d-glucoside	*S. buddleifolium*	Stem		[79]
419	Alangilignoside C	*S. buddleifolium*	Stem		[79]
420	(+)-(7*S*,8*R*,7′*E*)-4-Hydroxy-3,5,5′,9′-tetram ethoxy-4′,7-epoxy-8,3′-neo-lign-7′-en-9-ol	*S. erianthum*	Stem		[480]
421	(-)-(7*R*,8*S*,7′*E*)-4-Hydroxy-3,5,5′,9′-tetramethoxy-4′,7-epoxy-8,3′-neo-lign-7′-en-9-ol	*S. erianthum*	Stem		[480]
422	Liriodendrin	*S. lyratum*	Whole		[517]
423	Syringaresinol	*S. lyratum*	Whole		[517]
		*S. nigrum*	Whole		[496]
		*S. surattense*	Whole		[518]
424	Melongenamide A	*S. melongena*	Root		[210]
425	Cannabisin D	*S. melongena*	Root	Anti-inflammatory	[210]
426	Melongenamide B	*S. melongena*	Root	Anti-inflammatory	[210]
427	Grossamide	*S. melongena*	Root	Anti-inflammatory	[210]
428	Melongenamide C	*S. melongena*	Root	Anti-inflammatory	[210]
429	Cannabisin F	*S. melongena*	Root	Anti-inflammatory	[210]
430	Melongenamide D	*S. melongena*	Root	Anti-inflammatory	[210]
431	Cannabisin G	*S. melongena*	Root	Anti-inflammatory	[210]
432	1,2-dihydro-6,8-dimethoxy-7-hydroxy-1-(3,5-dimethoxy-4-hydroxyphenyl)-*N*^1^,*N*^2^-bis-[2-(4-hydroxyphenyl)ethyl]-2,3-naphthalene dicarboxamide	*S. melongena*	Root		[210]
433	Sisymbrifolin	*S. sisymbriifolium*	Fruit		[519]
434	Grossamide K	*S. melongena*	Root		[210]
435	Pinoresinol	*S. nigrum*	Whole		[496]
436	Pinoresinol 4-*O*-*β*-d-glucoside	*S. nigrum*	Whole		[520]
437	Medioresinol	*S. nigrum*	Whole		[496]
		*S. torvum*	Stem		[436]
438	Syringaresinol-4′-*O*-*β*-d-glucoside	*S. nigrum*	Whole		[520]
439	Glycosmisic acid	*S. surattense*	Whole		[518]
440	Simulanol	*S. surattense*	Whole		[518]
441	Balanophonin	*S. surattense*	Whole		[518]
442	Ficusal	*S. melongena*	Root		[209]
443	Tribulusamide A	*S. surattense*	Whole		[518]
444	Clemastanin B	*S. torvum*	Fruit		[521]
	*Other alkaloids*				
445	Xylogranatinine	*S. cathayanum*	Stem		[477]
446	Cernumidine	*S. cernuum*	Leaf		[109, 111, 112]
447	Isocernumidine	*S. cernuum*	Leaf		[111]
448	Cernidine	*S. cernuum*	Leaf		[501]
449	Ethyl orotate	*S. cathayanum*	Stem		[103, 477]
450	3-Indolecarboxylic acid	*S. americanum*	Aerial		[49]
451	L-Valyl-l-isoleucyl-l-leucine	*S. asperum*	Fruit		[67]
452	2-Methyltetrahydro-*β*-carboline	*S. jabrense*	Aerial		[166]
453	Proline	*S. asperum*	Fruit		[67]
454	Acetamide	*S. schimperianum*	Aerial		[277]
455	Stearamide	*S. schimperianum*	Aerial		[277]
456	(6*E*, 9*E*)*N*,*N*-dimethyloctadeca-6,9-dienamide	*S. schimperianum*	Aerial		[277]
457	(2*E*)-3-(4-Hydroxyphenyl)-*N*-[(2*S*)-2-(4-hydroxyphenyl)-2-methoxyethyl]-2-propenamide	*S. torvum*	Aerial		[450]
458	4-Coumaroyltyramine	*S. buddleifolium*	Stem		[79]
		*S. cathayanum*	Stem		[522]
		*S. indicum*	Root		[163]
		*S. melongena*	Root		[209]
		*S. surattense*	Whole		[518]
		*S. torvum*	Aerial		[338]
		*S. lyratum*	Whole		[507]
459	*N*-*trans*-Feruloyltyramine	*S. buddleifolium*	Stem		[79]
		*S. cathayanum*	Stem		[522]
		*S. indicum*	Root		[163]
		*S. melongena*	Root	Antidiabetic	[209]
		*S. lyratum*	Whole		[507]
460	*N*-*trans*-Feruloylmethoxytyramine	*S. buddleifolium*	Stem		[79]
		*S. cathayanum*	Stem		[522]
461	*N*-*trans*-Caffeoyltyramine	*S. buddleifolium*	Stem		[79]
462	*N*-*trans*-Feruloyldopamine	*S. buddleifolium*	Stem		[79]
463	*N*-*trans*-Feruloyloctopamine	*S. cathayanum*	Stem		[522]
		*S. septemlobum*	Aerial		[523]
464	*N*-*trans*-*p*-coumaroyloctopamine	*S. americanum*	Aerial	Antidiabetic	[49]
		*S. torvum*	Aerial		[524]
465	*N*-*trans*-*p*-feruloyloctopamine	*S. americanum*	Aerial	Antidiabetic	[49]
466	*N*-*trans*-*p*-coumaroyltyramine	*S. americanum*	Aerial	Antidiabetic	[49]
		*S. melongena*	Root		
467	*N*-*trans*-*p*-feruloytyramine	*S. americanum*	Aerial	Antidiabetic	[49]
		*S. torvum*	Aerial		[524]
468	*N*-*cis*-*p*-Coumaroyltyramin*e*	*S. melongena*	Root		[209]
469	Caffeoylputrescine	*S. melongena*	Stem		[205]
470	3-(3,4-Dihydroxyphenyl)-*N*-[3-[[4-[[3-(3,4-dihydroxyphenyl)-1-oxo-2-propen-1-yl] amino]butyl]amino]propyl]-2-propenamide	*S. melongena*	Stem		[205]
471	Aurantiamide acetate	*S. torvum*	Aerial		[524]
472	*N*^1^,*N*^4^,*N*^8^-Tris(dihydrocaffeoyl) spermidine	*S. sessiliflorum*	Fruit		[525]
473	*N*-(4-Aminobutyl)-*N*-[3-[[3-(3,4-dihydroxyphenyl)-1-oxopropyl] amino]propyl]-3,4-dihydroxybenzenepropanamide	*S. sessiliflorum*	Fruit		[525]
474	*N*-(3-Aminopropyl)-*N*-[4-[[3-(3,4-dihydroxyphenyl)-1-oxopropyl] amino]butyl]-3,4-dihydroxybenzenepropanamide	*S. sessiliflorum*	Fruit		[525]
475	Soya-cerebroside I	*S. torvum*	Root		[435]
	*Sterols*				
476	Cilistol G	*S. capsicoides*	Leaf		[85]
477	Capsisteroid A	*S. capsicoides*	Leaf		[85]
478	Capsisteroid B	*S. capsicoides*	Leaf		[85]
479	Capsisteroid C	*S. capsicoides*	Leaf		[85]
480	Capsisteroid D	*S. capsicoides*	Leaf		[85]
481	Capsisteroid E	*S. capsicoides*	Leaf		[85]
482	Capsisteroid F	*S. capsicoides*	Leaf		[85]
483	*β*-Sitosterol	*S. cathayanum*	Stem		[477, 522]
		*S. anguvi*	Fruit		[34]
		*S. cornifolium*	Aerial		[472, 473]
		*S. dulcamara*	Fruit		[130]
		*S. elaeagnifolium*	Whole		[134, 504]
		*S. indicum*	Whole		[160]
		*S. lycopersicum*	Seed		[478]
		*S. melongena*	Aerial		[206, 439]
		*S. schimperianum*	Aerial		[278]
		*S. surattense*	Aerial		[518]
		*S. torvum*	Root		[526]
		*S. trilobatum*	Whole		[356]
		*S. xanthocarpum*	Fruit		[398]
484	Daucosterol	*S. cathayanum*	Stem		[522]
		*S. chrysotrichum*	Leaf		[120]
		*S. elaeagnifolium*	Whole		[504]
		*S. glabratum*	Whole		[140]
		*S. ligustrinum*	Aerial		[179]
		*S. septemlobum*	Aerial		[523]
		*S. torvum*	Root		[526]
		*S. violaceum*	Whole		[392]
485	Campesterol	*S. elaeagnifolium*	Seed		[134]
		*S. melongena*	Root		[439]
486	Cholesterol	*S. lycopersicum*	Seed		[478]
		*S. sessiliflorum*	Fruit		[285]
487	*γ*-Sitosterol	*S. lycopersicum*	Seed		[478]
488	7-Oxositosterol	*S. violaceum*	Aerial		[391]
489	(3*β*)-7-Hydroxystigmast-5-en-3-yl *β*-d-glucoside	*S. violaceum*	Whole		[392]
490	Stigmasterol	*S. cornifolium*	Aerial		[472, 473]
		*S. dulcamara*	Fruit		[130]
		*S. elaeagnifolium*	Whole		[134, 504]
		*S. lycopersicum*	Seed		[478]
		*S. melongena*	Aerial		[439]
		*S. septemlobum*	Aerial		[523]
		*S. surattense*	Aerial		[527]
		*S. xanthocarpum*	Fruit		[398]
491	Brassicasterol	*S. elaeagnifolium*	Seed		[134]
492	Poriferasterol monoglucoside	*S. glabratum*	Whole		[140]
493	7-Oxostigmasterol	*S. violaceum*	Aerial		[391]
494	*β*-stigmasteryl-3-*O*-*β*-d-6-palmityl) glucoside	*S. septemlobum*	Aerial		[523]
495	Clerosterol	*S. elaeagnifolium*	Seed		[134]
496	7-Sitoster-3*β*-ol	*S. elaeagnifolium*	Seed		[134]
497	(3*β*,5*α*)Cholest-7-en-3-ol	*S. lycopersicum*	Seed		[478]
498	Stigmasta-5,24(28)-dien-3-ol	*S. elaeagnifolium*	Seed		[134]
		*S. torvum*	Leaf		[318]
499	Avenasterol	*S. elaeagnifolium*	Seed		[134]
500	5,24-Stigmastadienol	*S. elaeagnifolium*	Seed		[134]
501	*γ*-Tocopherol	*S. lycopersicum*	Seed		[478]
		*S. villosum*	Leaf		[479]
502	Ergosterol	*S. lycopersicum*	Seed		[478]
503	Lanosterol	*S. lycopersicum*	Seed		[478]
504	Peroxyergosterol	*S. lyratum*	Stem		[486]
		*S. violaceum*	Aerial		[391]
505	9,11-Dehydroergosterol peroxide	*S. lyratum*	Stem		[486]
		*S. violaceum*	Aerial		[391]
506	Nigralanostenone	*S. nigrum*	Leaf		[528]
507	Tumacone A	*S. nudum*	Leaf		[242, 247]
508	Tumacone B	*S. nudum*	Leaf		[242, 247]
509	Tumacoside A	*S. nudum*	Leaf	Antiplasmodial	[242, 247]
510	Tumacoside B	*S. nudum*	Leaf	Antiplasmodial	[242, 247]
511	SN-1	*S. nudum*	Aerial	Antiplasmodial	[245]
512	SN-2	*S. nudum*	Aerial	Antiplasmodial	[245]
513	SN-3	*S. nudum*	Aerial	Antiplasmodial	[245]
514	SN-4	*S. nudum*	Aerial	Antiplasmodial	[245]
515	SN-5	*S. nudum*	Aerial	Antiplasmodial	[245]
516	9*α*,11*α*-epidioxyergosta-6,22-dien-3*β*-ol	*S. septemlobum*	Aerial		[523]
517	Carpesterol	*S. capsicoides*	Seed	Anticancer, antifungal	[86]
		*S. sisymbriifolium*	Fruit		[519]
518	Carpesterol methyl ether	*S. xanthocarpum*	Fruit	Antifungal	[401]
519	Carpesterol ethyl ether	*S. xanthocarpum*	Fruit	Antifungal	[401]
520	Stigmast-7-en-6-one, 3-*β*-d-glucosyloxy)22-hydroxy-4-methyl-(3*β*,4*α*,5*α*,22R)	*S. xanthocarpum*	Fruit	Antifungal	[401]
521	Stigmast-7-en-6-one, 3-*β*-d-glucosyloxy)22-methoxy-4-methyl-(3*β*,4*α*,5*α*,22R)	*S. xanthocarpum*	Fruit	Antifungal	[401]
522	Toptriol	*S. glaucophyllum*	Leaf		[529]
523	Cholecalciferol	*S. glaucophyllum*	Leaf		[530]
524	*β*-d-Glucoside, (1α,3*β*,5*Z*,7*E*)-3,25-dihydroxy-9,10-secocholesta -5,7,10(19) –trien -1-yl	*S. glaucophyllum*	Leaf		[530]
525	Dehydrocholesterol				
526	3,4-Dihydro-3,5,8-trimethyl-3-(4,8,12-trimethyltridecyl)-2*H*-1-benzopyran-7-yl acetate	*S. villosum*	Leaf		[479]
527	Tumaquenone	*S. nudum*	Aerial		[247]
528	Abutiloside A	*S. abutiloides*	Root		[5, 7–9]
529	Abutiloside B	*S. abutiloides*	Root		[5]
530	Abutiloside H	*S. abutiloides*	Root		[5]
531	Abutiloside I	*S. abutiloides*	Root		[5]
532	Abutiloside J	*S. abutiloides*	Root		[5]
533	Abutiloside K	*S. abutiloides*	Root		[5]
534	Abutiloside C	*S. abutiloides*	Root		[7, 8]
535	Abutiloside D	*S. abutiloides*	Root		[6]
536	Abutiloside E	*S. abutiloides*	Root		[6]
537	Abutiloside F	*S. abutiloides*	Root		[6]
538	Abutiloside G	*S. abutiloides*	Root		[6]
539	Aethioside A	*S. aethiopicum*	Stem		[28]
540	Aethioside B	*S. aethiopicum*	Stem		[28]
541	Aethioside C	*S. aethiopicum*	Stem		[28]
	*Phenolic compounds*				
542	4-Caffeoylquinic acid	*S. melongena*	Stem,Leaf		[205, 531]
		*S. lyratum*	Whole		[517]
543	5-Caffeoylquinic acid	*S. melongena*	Stem		[205]
		*S. sessiliflorum*	Fruit		[525]
544	(1*R*,3*R*,4*S*,5*R*)-3-(Acetyloxy)-5-[[(2*E*)-3-(3,4-dihydroxyphenyl)-1-oxo-2-propen-1-yl]oxy] -1,4-dihydroxycyclohexanecarboxylic acid	*S. melongena*	Stem		[205]
545	(1*S*,3*R*,4*R*,5*R*)-3-(Acetyloxy)-4-[[(2*E*)-3-(3,4-dihydroxyphenyl)-1-oxo-2-propen-1-yl]oxy] -1,5-dihydroxycyclohexanecarboxylic acid	*S. melongena*	Stem		[205]
546	Chlorogenic acid	*S. anguvi*	Fruit	Anticancer	[31]
		*S. guaraniticum*	Leaf		[146]
		*S. incanum*	Aerial		[494]
		*S. lycocarpum*	Fruit		[532]
		*S. lyratum*	Whole		[517]
		*S. melongena*	Stem,Leaf		[205, 531]
		*S. surattense*	Whole		[99]
547	Neochlorogenic acid	*S. lyratum*	Whole		[517]
548	Rosmarinic acid	*S. betaceum*	Fruit		[78]
		*S. guaraniticum*	Leaf		[146]
549	3,5-Dicaffeoylquinic acid	*S. melongena*	Stem		[91]
550	(*Z*)-Neochlorogenic acid	*S. melongena*	Stem		[91]
551	Gallic acid	*S. anguvi*	Fruit	Anticancer	[31]
		*S. cernuum*	Leaf		[112]
		*S. spirale*	Aerial		[299]
		*S. surattense*	Whole		[99]
552	4-hydroxybenzoic acid	*S. crinitum*	Fruit		[122]
		*S. americanum*	Aerial		[49]
553	Protocatechuic acid	*S. lyratum*	Whole		[514]
		*S. spirale*	Leaf		[297]
		*S. nigrum*	Whole		[520]
554	Vanillic acid	*S. lyratum*	Whole		[514]
		*S. sessiliflorum*	Fruit		[510]
		*S. nigrum*	Whole		[520]
		*S. vestissimum*	Fruit		[491]
555	Caffeic acid	*S. anguvi*	Fruit	Anticancer	[31]
		*S. guaraniticum*	Leaf		[146]
		*S. incanum*	Aerial		[506]
		*S. lycocarpum*	Fruit		[532]
		*S. lyratum*	Whole		[194]
		*S. melongena*	Stem		[205]
		*S. muricatum*	Whole		[215]
		*S. surattense*	Whole		[99, 518]
		*S. xanthocarpum*	Root		[427]
556	*P*-Coumaric acid	*S. americanum*	Aerial		[49]
557	Isoferulic acid	*S. cernuum*	Leaf		[109, 112]
558	2,4,6-Trimethoxyphenol	*S. torvum*	Stem		[533]
559	Propionylsyringol	*S. torvum*	Stem		[533]
560	Resveratrol	*S. americanum*	Fruit		[45]
561	*cis*-*p*-Coumaric acid ethyl ester	*S. crinitum*	Fruit		[122]
562	*cis*-*p*-Coumaric acid	*S. crinitum*	Fruit		[122]
563	*trans*-*p*-Coumaric acid ethyl ester	*S. crinitum*	Fruit		[122]
564	*trans*-*p*-Coumaric acid	*S. crinitum*	Fruit		[122]
		*S. incanum*	Aerial		[506]
565	Erythro-1,2-bis-(4-hydroxy-3-methoxyphenyl)-1,3-propanediol	*S. lyratum*	Whole		[517]
566	Threo-1,2-bis-(4-hydroxy-3-methoxyphenyl)-1,3-propanediol	*S. lyratum*	Whole		[517]
567	Evofolin B	*S. surattense*	Whole		[518]
568	Ethyl caffeate	*S. nienkui*	Whole		[509]
569	Methyl salicylate	*S. nienkui*	Whole		[509]
		*S. aculeastrum*	Leaf		[11]
570	*p*-Hydroxybenzoic acid	*S. nigrum*	Whole		[520]
571	Vanillin	*S. nienkui*	Whole		[509]
572	Protocatechuic aldehyde	*S. nienkui*	Whole		[509]
573	3,5-Diethoxyphenol	*S. nigrum*	Leaf		[528]
574	Quinic acid	*S. sessiliflorum*	Fruit		[525]
575	Phenol	*S. sessiliflorum*	Fruit		[525]
576	Salicylic acid	*S. torvum*	Aerial		[524]
577	Violaxanthin	*S. sessiliflorum*	Fruit		[525]
578	Lutein	*S. sessiliflorum*	Fruit		[525]
579	*α*-Carotene	*S. sessiliflorum*	Fruit		[525]
580	Kryptoxanthin	*S. sessiliflorum*	Fruit		[525]
581	Luteoxanthin	*S. sessiliflorum*	Fruit		[525]
582	15-cis-*β*-Carotene	*S. sessiliflorum*	Fruit		[525]
583	Foliaxanthin	*S. sessiliflorum*	Fruit		[525]
584	Physoxanthin	*S. sessiliflorum*	Fruit		[525]
585	Coniferol	*S. surattense*	Whole		[518]
586	1,2-Bis(4-hydroxy-3-methoxyphenyl)-1,3-propanediol	*S. surattense*	Whole		[518]
587	Threo-1-(4-Hydroxy-3-methoxyphenyl)-2-[4-[(*E*)-3-hydroxy-1-propenyl]-2-methoxy phenoxy]-1,3-propanediol	*S. surattense*	Whole		[518]
588	Tyrosol C	*S. validinervium*	Aerial		[534]
589	(*E*)-Coniferaldehyde	*S. melongena*	Root		[209]
590	*trans*-Cinnamic acid	*S. spirale*	Leaf	Antibacterial	[297]
591	Methyl caffeate	*S. torvum*	Fruit	Antibacterial,antidiabetic	[315, 320, 335–337]
592	(*E*)-2,3-dihydroxycyclopentyl-3-(3′,4′-dihydroxyphenyl)acrylate	*S. torvum*	Fruit	Antihypertensive	[521]
593	Eugenol	*S. torvum*	Stem		[533]
	*Coumarins and coumestans*				
594	Scopolin	*S. cathayanum*	Stem	Anticancer	[104, 105]
		*S. lyratum*	Whole		[194]
		*S. septemlobum*	Aerial		[523]
595	Scopoletin	*S. glabratum*	Whole		[140]
		*S. indicum*	Seed		[535]
		*S. ligustrinum*	Aerial		[179]
596	Coumarin	*S. incanum*	Leaf		[494]
		*S. surattense*	Whole		[99]
		*S. vestissimum*	Fruit		[491]
597	Fraxetin	*S. indicum*	Seed		[536]
598	Isofraxidin	*S. indicum*	Seed		[536]
599	Umbelliferone	*S. lycopersicum*	Aerial		[438]
600	7-hydroxy-6,8-dimethoxy-3-(4′-hydroxy-3′-methoxyphenyl)-coumarin	*S. indicum*	Seed		[536]
601	Cleosandrin	*S. indicum*	Seed		[535]
602	4,4′-Biisofraxidin	*S. indicum*	Seed		[535]
603	Arteminorin A	*S. indicum*	Seed		[535]
604	Indicumin E	*S. indicum*	Seed		[536]
605	Bergaptin	*S. lycopersicum*	Aerial		[438]
606	Aesculetin	*S. lycopersicum*	Aerial		[438]
		*S. validinervium*	Aerial		[534, 537]
607	6, 7-Dimethoxycoumarin	*S. melongena*	Root		[209]
608	Escopoletin	*S. nigrum*	Whole		[520]
609	Isoscopoletin	*S. validinervium*	Aerial		[534, 537]
610	1′-*O*-7-esculetin-4′-*O*-1″-ethylenglycol-*β*-d-glucose	*S. validinervium*	Aerial		[534]
611	Coumestrol	*S. lyratum*	Whole	Anti-inflammatory	[88]
612	9-hydroxy-2′,2′-dimethyl[5′,6′:2,3]-coumestan	*S. lyratum*	Whole	Anti-inflammatory	[88]
613	Solalyratin A	*S. lyratum*	Whole	Anti-inflammatory	[88]
	*Coumarinolignoids*				
614	Indicumine A	*S. indicum*	Seed	Anti-HBV	[535]
615	Indicumine B	*S. indicum*	Seed	Anti-HBV	[535]
616	Indicumine C	*S. indicum*	Seed		[535]
617	Indicumine D	*S. indicum*	Seed		[535]
	*Fatty acids and esters*				
618	Hexadecanoic acid	*S. aculeastrum*	Leaf		[11]
		*S. vestissimum*	Fruit		[490]
		*S. villosum*	Leaf		[434, 479]
619	Octadecanoic acid,	*S. aculeastrum*	Leaf		[11]
		*S. erianthum*	Leaf		[137]
620	Linoleic acid	*S. aculeastrum*	Leaf		[11]
		*S. glabratum*	Whole		[140]
621	Lignoceric acid	*S. cathayanum*	Stem		[477]
622	Corchorifatty acid B	*S. americanum*	Aerial		[49]
623	Linolenic acid	*S. erianthum*	Leaf		[137]
		*S. glabratum*	Whole		[140]
624	9(*Z*),11(*E*)-Octadecadienoic acid	*S. erianthum*	Leaf		[137]
625	13*S*-Hydroxy-9(*Z*),11(*E*)-octadecadienoic acid	*S. erianthum*	Leaf		[137]
626	9*S*-Hydroxy-10(*E*),12(*Z*),15(*Z*)-octadecatrienoic acid	*S. erianthum*	Leaf		[137]
627	Decosahexaenoic acid	*S. glabratum*	Whole		[140]
628	Decosapentaenoic acid	*S. glabratum*	Whole		[140]
629	Oleic acid	*S. glabratum*	Whole		[140]
630	Eicosapentaenoic acid	*S. glabratum*	Whole		[140]
631	Lauric acid	*S. glabratum*	Whole		[140]
632	Palmitoleic acid	*S. glabratum*	Whole		[140]
633	Arachidonic acid	*S. glabratum*	Whole		[140]
		*S. trilobatum*	Whole		[356]
634	Myristic acid	*S. glabratum*	Whole		[140]
635	Gamma-linolenic acid	*S. glabratum*	Whole		[140]
636	9-Oxo-(10*E*, 12*Z*)-octadecadienoic acid	*S. melongena*	Calyx		[91]
637	(10*Z*,12*E*)-9-Oxo-10,12-octadecadienoic acid	*S. melongena*	Calyx		[91]
638	Eicosanoic acid	*S. torvum*	Root		[526]
639	Octacosanoic acid	*S. torvum*	Root		[526]
640	4-(3,5-Di-Tert-Butyl-4-Hydroxy Phenyl) butyl Acrylate	*S. villosum*	Leaf		[479]
	*Others*				
641	Puerariafuran	*S. lyratum*	Whole	Anti-inflammatory	[88]
642	1,2-Benzenedicarboxylic acid	*S. aculeastrum*	Leaf		[11]
643	1, 4-Dimethyl-benzene	*S. aculeastrum*	Leaf		[11]
644	*n*-Nonane	*S. aculeastrum*	Leaf		[11]
645	*n*-Octanol	*S. aculeastrum*	Leaf		[11]
646	Methyl hexadecanoate	*S. aculeastrum*	Leaf		[11]
647	Dodecane	*S. aculeastrum*	Leaf		[11]
648	Undecanal	*S. aculeastrum*	Leaf		[11]
649	Nonanal	*S. aculeastrum*	Leaf		[11]
650	Eicosane	*S. aculeastrum*	Leaf		[11]
		*S. betaceum*	Fruit		[77]
651	Methyl-9,12-octadecadienoate	*S. aculeastrum*	Leaf		[11]
652	Hexadecane	*S. aculeastrum*	Leaf		[11]
653	9,17-Octadecadienal	*S. aculeastrum*	Leaf		[11]
654	Hexanal	*S. betaceum*	Fruit		[78]
655	Ethyl butanoate	*S. betaceum*	Fruit		[78]
656	4-Hydroxy-4-methyl-2-pentanone	*S. betaceum*	Fruit		[78]
657	2,3-Butanediol	*S. betaceum*	Fruit		[78]
658	*cis*-3-Hexen-1-ol	*S. betaceum*	Fruit		[78]
659	3(*Z*)-Hexenal	*S. betaceum*	Fruit		[78]
660	Ethyl-*α*-d-arabinofuranoside	*S. lyratum*	Whole		[514]
661	Solalyratin B	*S. lyratum*	Whole	Anti-inflammatory	[88]
662	1-{1-[2-(2 hydroxypropoxy) propoxy] propan-2-yloxy} propan-2-ol	*S. schimperianum*	Aerial		[277]
663	5-Hydroxymethyl furfural	*S. torvum*	Stem		[533]
664	Solanesol	*S. tuberosum*	Leaf		[538]
665	3-Hydroxymethyl-7-methoxywutaifuranol	*S. cathayanum*	Whole		[102]
666	Phenylmethyl 2-*O*-*β*-d-xylosyl-*β*-d-glucoside	*S. incanum*	Aerial		[506]
667	Zizybeoside I	*S*. *lycopersicum*	Fruit		[511]
668	Methyl salicylate 2-*O*-*β*-d-glucosyl-(1-2)-[O-*β*-d-xylosyl-(1-6)]-*O*-*β*-d-glucoside	*S*. *lycopersicum*	Fruit		[511]
669	Phenethyl alcohol 8-*O*-*β*-d-glucosyl-(1-2)-[O-*α*-l-arabinosyl-(1-6)]-*O*-*β*-d-glucoside	*S*. *lycopersicum*	Fruit		[511]
670	Benzyl alcohol 7-*O*-*β*-d-glucosyl-(1-2)-[*O*-α-l-arabinosyl-(1-6)]-*β*-d-glucoside	*S*. *lycopersicum*	Fruit		[511]

### Steroidal Saponins

**Fig. 1:**
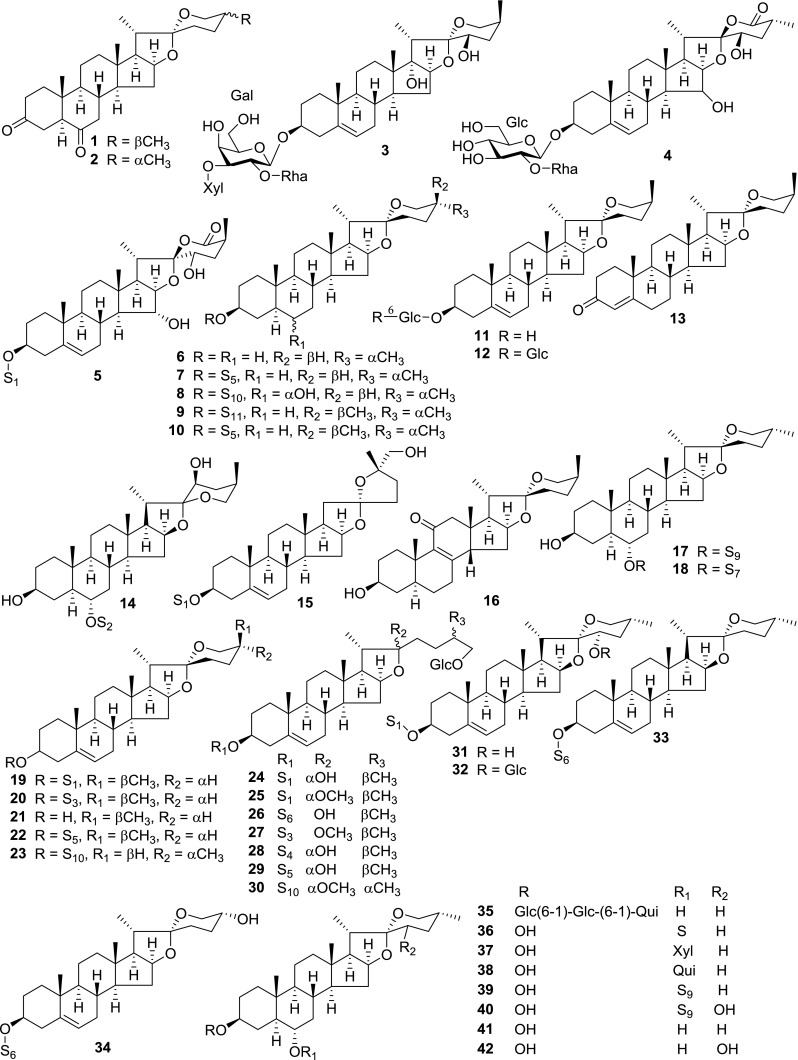

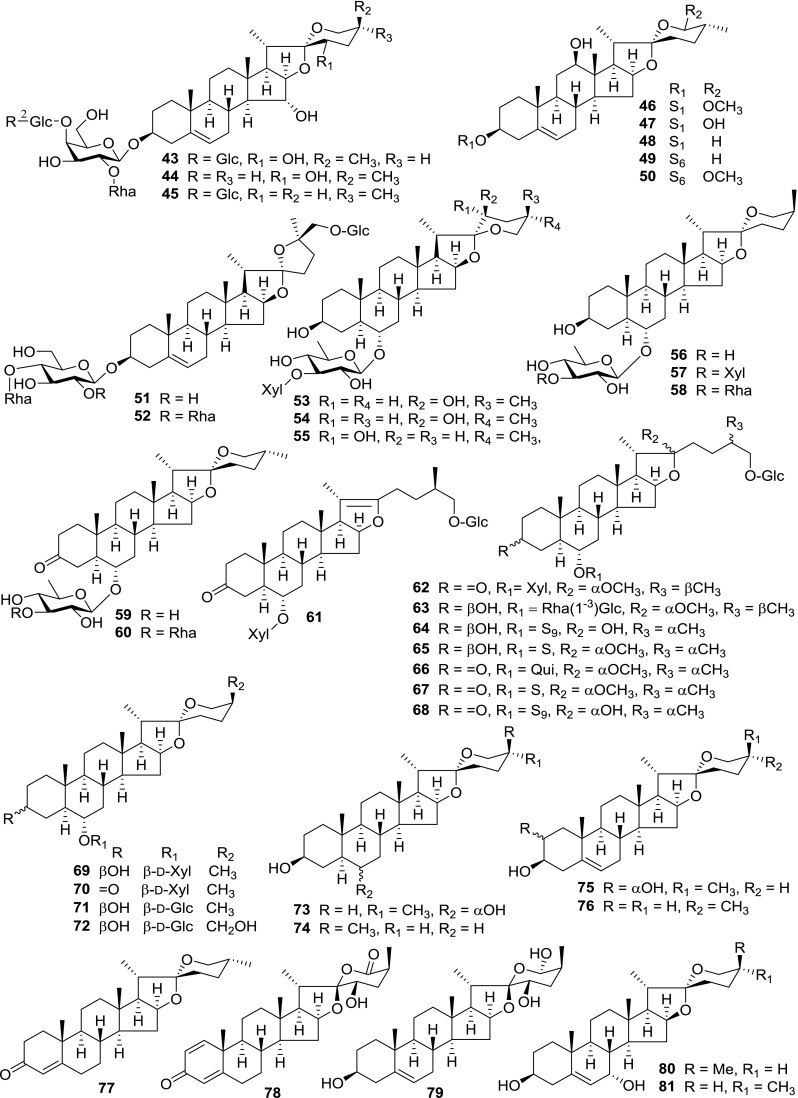

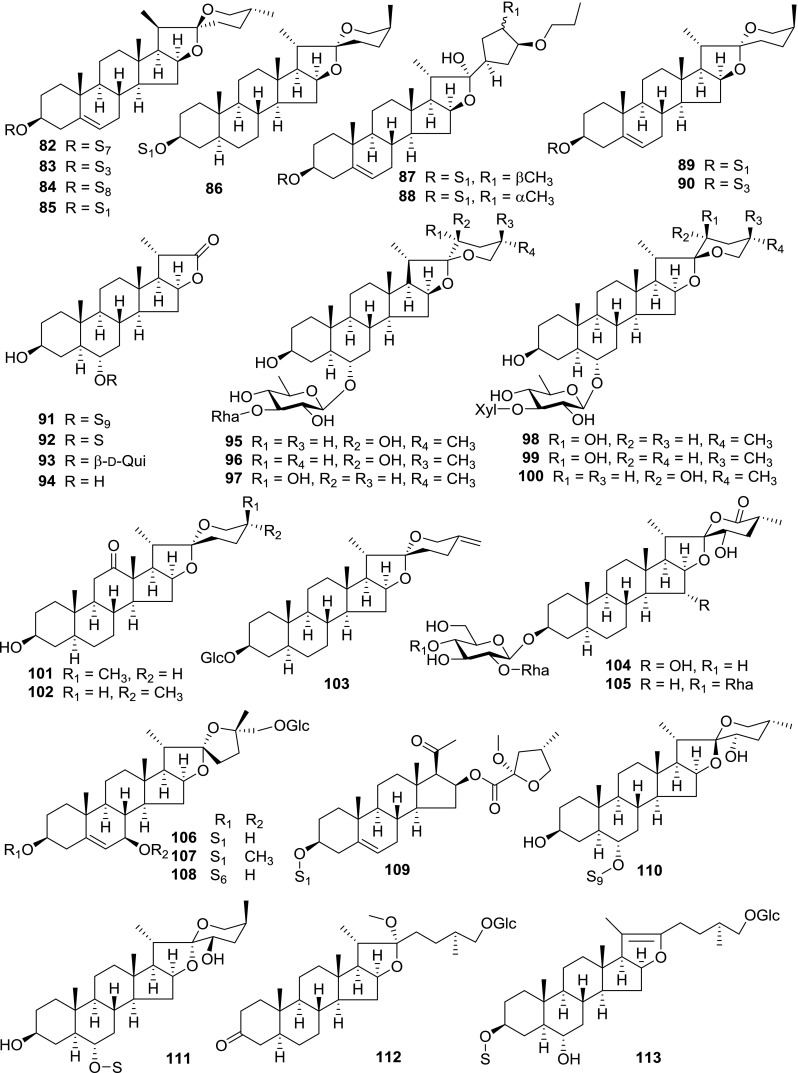

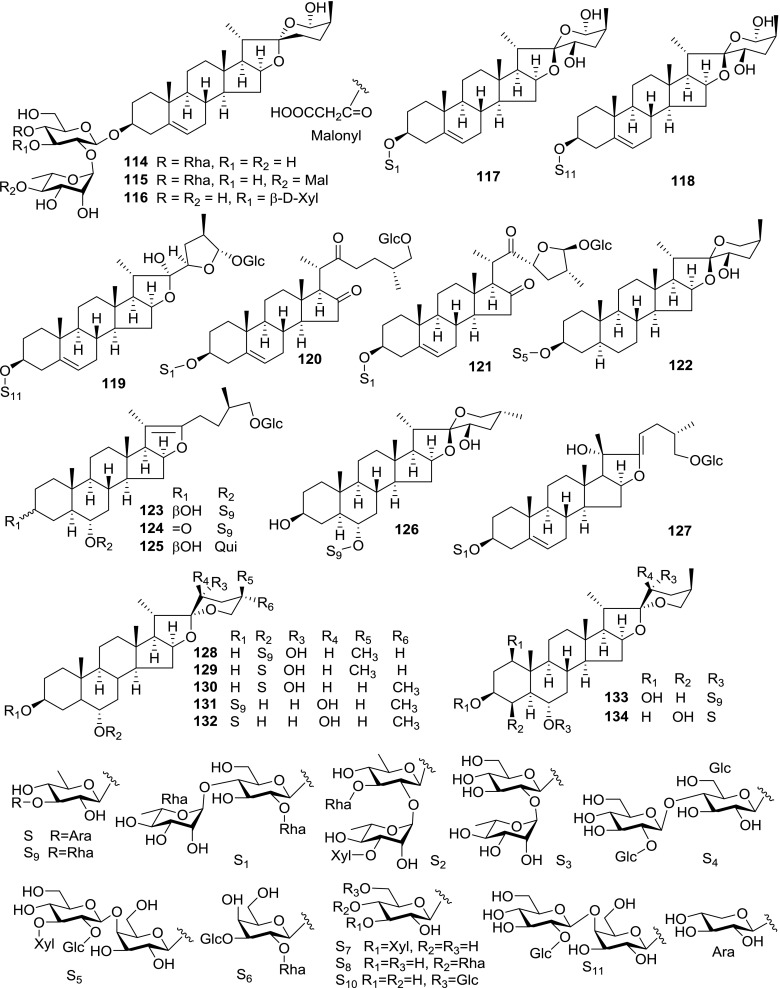
Steroidal saponins **1**–**134** from *Solanum*

Steroidal saponins are prominent characteristic components in *Solanum* species, from which 134 compounds, **1**–**134**, have been obtained (Fig. 1). Among all the studied species, *S. torvum* was the one studied mostly, resulting in the isolation of 32 saponins including chlorogenone (**1**), (5*α*,25*S*)-spirostan-3,6-dione (**2**), diosgenone (**13**), **56**–**72**, neochlorogenin (**73**), solanolactosides A–C (**91**–**93**), torvosides J–L (**95**–**97**) and **98**–**102** from the leaves, fruits, aerial parts and the whole plant [323, 325, 430, 435, 436, 448, 449, 451, 452, 463].

Included herein are spirostane saponins, SC1–SC6 (**35**–**40**), isolated from the leaves of *S. chrysotrichum* [113–115, 117], and lyconosides Ia (**46**), Ib (**47**), II (**48**), III (**49**), and IV (**50**) reported from the fruits of *S. lycocarpum*. Indiosides G (**82**) and H (**83**) with an iso-type F ring were isolated from the methanolic extract of the whole plant of *S. violaceum*, together with indioside I (**86**), and two unusual furostanol saponins with a deformed F ring, indiosides J (**87**) and K (**88**) [391, 392]. In addition, four steroidal sapogenins, indiosides L–O (**78**–**81**) were also obtained from this plant [391]. Indioside L (**78**) is a rare spirostanoid possessing a 1,4-dien-3-one moiety in ring A. Compounds **80** and **81** represent rare examples of spirostane with the 3*β*,7*α*-diol-5,6-ene moiety compared to the normal 3*β*,7*β*-diol-5,6-ene derivatives [391].

Two C-22 steroidal lactone saponins, namely solanolactosides A, B (**91**, **92**) and two spirostanol glycosides, torvosides M, N (**23**, **8**) were isolated from ethanol extract of aerial parts of *S. torvum.* Compounds **91** and **92** possess the aglycon of solanolide (**94**), while **23** and **8** have the aglycons of yamogenin (**76**) and neochlorogenin (**73**), resp. The aglycon of **94** is an unusual C-22 steroidal lactone sapogenin [316].

An avenacoside-type saponin (**51**) was isolated from aerial parts of *S. surattense* [305]. Two 23-keto-spirostanol glycosides, torvoside Q (**18**) and paniculonin B (**126**) were obtained from aerial parts of *S. torvum* [323, 331]. Torvosides A (**64**), B (**65**), F (**67**) and G (**112**) displayed a positive reaction with Ehrlich reagent, suggesting these to be furostanol glycosides [449]. Abutilosides L (**106**), M (**107**) and N (**108**), a 22*S*,25*S*-epoxy-furost-5-ene type glycosides, and abutiloside O, being a 20,22-*seco*-type steroidal glycoside, were isolated from the fresh fruits of *S. abutiloides* [4].

Anguiviosides III (**118**) and XI (**119**) are hydroxylated at C-23 and C-26 on the spirostanol and furostanol skeletons, resp. Anguiviosides XV (**120**) and XVI (**121**) are based on a 16, 22-dicarbonyl aglycon, with **121** hydroxylated at C-23 and C-26 followed by ring closure. The biogenetic pathway of 16,22-dicarbonyl compounds such as **120** and **121** might be considered via a 17*R*-hydroxy spirostanol such as pennogenin, 11 or via a 3*β*,16*β*,22,26-tetrahydroxycholesterol glycoside such as anguivioside A (**114**) [43].

*Solanum* saponins were reported to have various bioactivies, e.g. cytotoxic [257], anticancer [316, 317, 392], hepatoprotective [242, 247], antihypertensive [289, 291], antimelanogenesis [211], antifungal [113, 114, 117], anti-inflammatory [331, 392, 448] anticonvulsant [305] and antiviral [257].

Nuatigenosido (**15**) from the roots of *S. sisymbriifolium* presented anti-hypertensive effect in experimental hypertensive rats [291]. Dioscin (**19**) showed antimelanogenesis effect on α-melanocyte stimulating hormone (α-MSH)induced melanogenesis in B16 murine melanoma cells. It significantly downregulated the expression of tyrosinase, TRP-1, and TRP-2, which led to the reduction of α-MSH-induced melanogenesis in B16 cells [211]. Degraded diosgenone (**13**) from *S. nudum* exhibited hepatoprotective effect on the liver of mice infected with *Plasmodium berghei*; necrosis of hepatocytes in mice infected with malaria decreased 47–65 [249].

Spirostanic saponins SC2-SC6 (**36**–**40**) from the leaves of *S. chrysotrichum* displayed activity against dermatophytes and yeasts. **36** was the most active in indicating fungicidal effect against *Candida albicans* and non-albicans strains [113, 114, 117].

Indioside H (**83**), borassoside E (**85**), indioside I (**86**) and yamoscin (**89**) demonstrated cytotoxic activity against six human cancer cell lines (HepG2, Hep3B, A549, Ca9-22, MDA-MB-231, and MCF-7) (IC_50_ = 1.83–8.04 μg/mL) [392]. Seperately, **85** and **86** presented inflammation inhibitory effects on SAG (IC_50_ = 0.62 ± 0.03 and 2.84 ± 0.18 μg/mL, resp.). Compound **85** also inhibited elastase release with IC_50_ values of 111.05 ± 7.37 μg/mL [392], while **89** showed anti-neutrophilic inflammatory activity against SAG with an IC_50_ value of 3.59 μM [331].

Torvosides N (**8**) and M (**23**) revealed significant cytotoxicity against MGC-803, HepG2, A549 and MCF-7 as compared to the positive control, CDDP [316]. Torvosides J-L (**95**–**97**), isolated from the leaves of *S. torvum*, exhibited substantial anticonvulsant activity in zebrafish seizure assays [323], while **96** also showed considerable antifungal activity against *Aspergillus flavus* and *Fusarium verticillioides* with MIC ranging from 31.25 to 250 μg/mL [318]. Compounds **99** and **100** inhibited both inflammatory mediators SAG (IC_50_ = 3.49 and 2.87 μM) and elastase release (IC_50_ = 2.69 and 0.66 μM) [331], while **123**–**125** convinced cytotoxicities against melanoma A375 [317].

### Steroidal Alkaloids

**Fig. 2:**
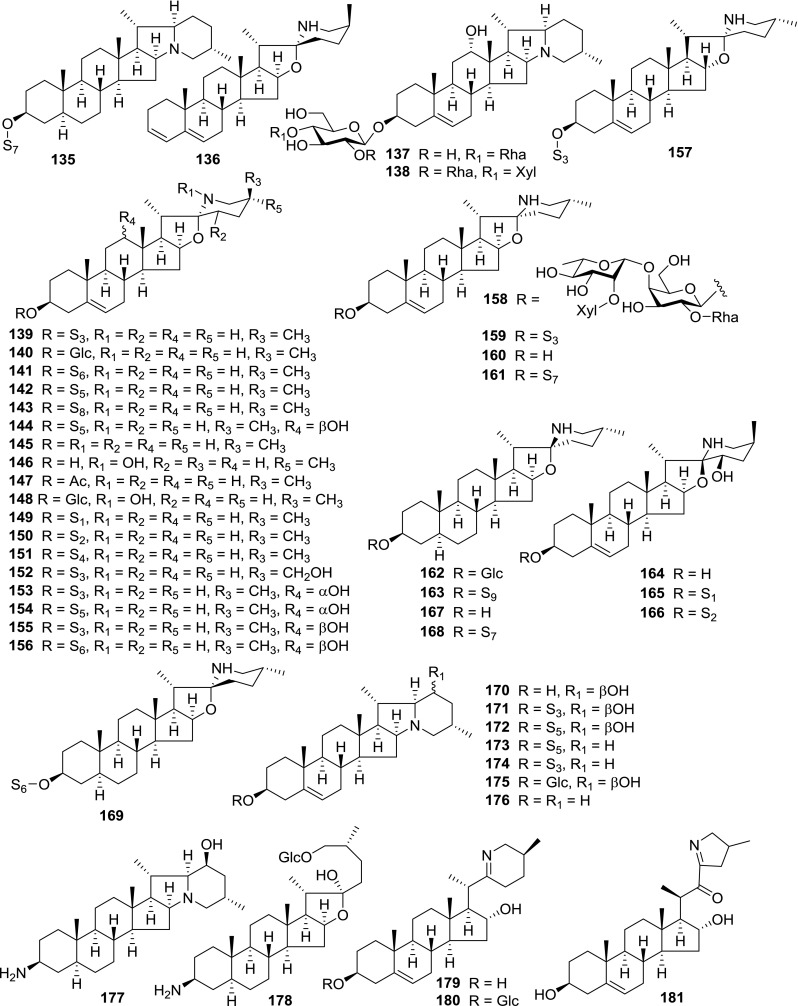

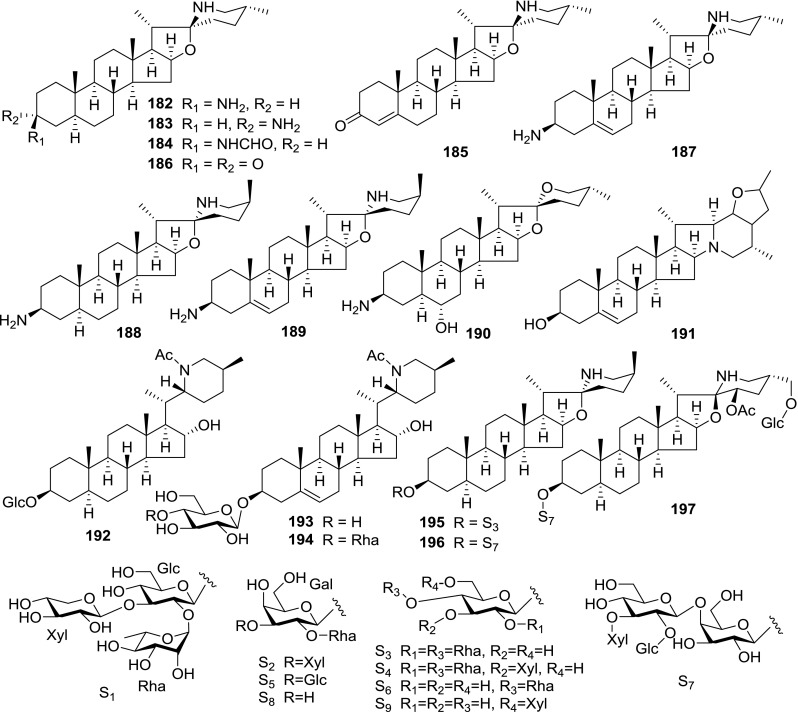
Steroidal alkaloids **135**–**197** from *Solanum*

Sixty-three steroidal alkaloids (**135**–**197**), as other principal components in *Solanum* were reported from this genus (Fig. 2). Compounds **139**–**156** are derivatives of solasodine (**145**), one of the main glycoalkaloid constituents in *Solanum* spp., even as indicated by several numbers of species from which it has been isolated. Solamargine (**139**) is the major steroidal alkaloid constituent of *Solanum* plants and literature data showed that it has been revealed in 18 species.

Compounds such as **139**, solasonine (**142**), β1-solasonine (**143**) and solanigroside P (**156**) with three sugar units and α-l-rhamnose at C-2 or a hydroxyl group on the steroidal backbone may be potential candidates for the treatment of gastric cancer [228].

Featured here are steroidal pseudoalkaloid oligoglycosides, robeneosides A (**153**) and B (**154**) and lobofrutoside (**155**) from the fruits of *S. lycocarpum* [182, 447], and a rare 16*β*-H steroidal alkaloid (**157**) from aerial parts of *S. surattense* [305]. Also included are leptinine I (**171**) and II (**172**), the solanidane alkaloid glycosides, isolated from aerial parts of *S. orbignianum* [46].

Two rare C-3 amino steroidal alkaloids, **188** and **189**, were isolated from aerial parts of *S. triste* [362, 471]. Three C-27 steroidal glycoalkaloids, spiralosides A (**194**), B (**193**), C (**192**), were obtained from the fruits of *S. spirale* [474]. Esculeoside A (**197**), a tomato saponin, is a significant component of ripened tomatoes isolated by Toshihiro et al. [475].

Various bioactivities e.g. antibacterial [80, 384, 403, 406, 407], anticancer [13, 305, 458], antidiabetic [182, 183], antifungal [279], anti-inflammatory [303], CNS depressant [294], leishmanicidal [182, 183], molluscicidal [384, 403, 406, 407], neurotoxicity [106], schistosomicidal [185, 186, 447, 457], spasmolytic [70] and trypanocidal [185, 186, 447, 457] were highlighted as have been exhibited by steroidal alkaloids of *Solanum*.

Antioxidant activity of **145** and tomatidine (**167**) from the berries of *S. aculeastrum* was investigated using DPPH, ABTS and reducing power assays, and the highest inhibition was observed when the two compounds were combined, followed by **145** and **167** [13]. Furthermore, **145** exhibited significant anti-inflammatory activity at doses of 30 mg/kg, with a maximum inhibition of 77.75% in carrageenan-induced rat paw edema, comparing to indomethacin (81.69%). It also showed stronger (46.79effect in xylene induced ear edema in mice [303]. Intraperitoneal injection of **145** (25 mgkg) significantly delayed latency of hind limb tonic extensor phase in the picrotoxin-induced convulsions, and it also potentiated thiopental-provoked sleep in a dose-dependent manner [294]. Moreover, **145** exhibited not only the antibacterial activity against *Klebsiella* and *Staphylococcus* spp. at concentration of 1 mg, together with **139** and **141** [403], but also a potent stemness and invasion inhibitory effect on human colorectal cancer HCT116 cells [155]. Colony Spheroid formation assay showed that solasodine dose-dependently prohibited HCT116 cell stemness. CD133, CD44, Nanog, Oct-4 and Sox-2 were inhibited by **145** to reverse stemness and similar mechanism was stimulated in vivo. Transwell and scratch wound assays revealed that **145** impeded HCT116 cell invasion and migration potential strengthened by TGF-β1. Moreover, solasodine attenuated TGF-β1-induced EMT and decreased MMPs while in vivo study showed the same trend. The results of this study implied that **145** may be a novel therapeutic drug for CRC treatment [155].

Burger et al. documented that the crude extract and aqueous fraction containing **139** displayed potent non-selective cytotoxicity (IC_50_ 15.62 μgmL) and noteworthy 9.1-fold P-glycoprotein inhibition at 100 μgmL [15]. Zhang et al. assessed the molecular mechanism underlying the anti-cancer effect of **139** in human cholangiocarcinoma QBC939 cells. The results revealed that **139** inhibited the viability of QBC939 cells in a dose-dependent manner. Furthermore, it significantly induced the apoptosis of QBC939 cells and altered the mitochondrial membrane potential of cells. Quantitative polymerase chain reaction analysis revealed that **139** decreased the mRNA level of B cell lymphoma-2 (Bcl-2) Bcl-extra-large and X-linked inhibitor of apoptosis protein but increased the mRNA level of Bcl-2-associated X protein (Bax) In addition, western blot analysis demonstrated that **139** inhibited the protein expression of Bcl-2 and poly ADP ribose polymerase (PARP) and promoted the protein expression of Bax, cleaved PARP, caspase 3, cleaved caspase 3 and caspase [97].

Compounds **139**, **141** and **157** demonstrated cytotoxicity against A549, whereas **139** and **156** showed cytotoxicity against HepG2 cell lines [305]. Compounds **139** and **141** were confirmed as the effective components for *Oncomelania* snail control. The death rate of *Oncomelania* snails was 94.2 at a concentration of 2.50 mg/L (**139**) [406], while **141** exhibited a lethality of 100against *O. hupensis* [407]. Moreover, **139** and solasonine (**142**) displayed not only leishmanicidal activity against promastigote forms of *Leishmania amazonensis* [185], but also antidiabetic activity by inhibiting the serum glucose increase in oral sucrose-loaded rats and suppressing gastric emptying in mice [182]. A synergistic effect was observed for a mixture of the compounds [183]. Compound **139** also expressed stronger trypanocidal activity (IC_50_ = 15.3 μg/mL), when compared to benznidazol (IC_50_ = 9.0 μg/mL), the only drug used to treat Chagas’ disease [186].

Tomatine (**168)** was illustrated to exert significant neuroprotective effect on H_2_O_2_-induced SH-SY5Y cells, by enhancing intracellular anti-oxidant enzyme activity and brain-derived neurotrophic factor expression and restraining H_2_O_2_-induced oxidative stress [106]. Isojuripidine (**190**) displayed spasmolytic activity by hindering phasic contractions induced by both histamine and acetylcholinein guinea-pig ileum [69].

### Pregnane Glycosides

**Fig. 3:**
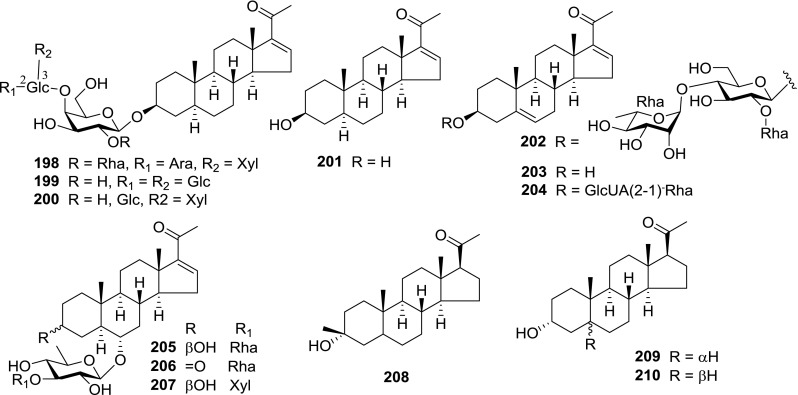
Pregnane glycosides **198**–**210** from *Solanum*

Compounds **198**–**210** from *Solanum* comprise pregnane glycosides (Fig. 3). These compounds coexist in small amounts and could be biosynthesised from steroidal glycosides [194]. Solanigrosides A (**198**), B (**199**), **200** and hypoglaucin H (**202**) were isolated from *S. nigrum* [476]. Aerial parts of *S. torvum* gave the highest number of pregnane glycosides, torvpregnanosides A (**205**) and B (**207**), ganaxolone (**208**), allopregnanolone (**209**) and pregnanolone (**210**). The whole plant of *S. lyratum* afforded compounds **203** and **204** [194].

Pregnane glycosides have reportedly demonstrated anticancer properties [194, 317]. Compound **203** exhibited substantial cytotoxic activity against A375-S2, HeLa, SGC-7901, and Bel-7402 cell lines, with IC_50_ values of 13.1 to 49.8 μg/mL [194]. Compound **206** indicated cytotoxicity against human melanoma A375 (IC_50_ = 39.66 μM) [317].

### Triterpenes

**Fig. 4:**
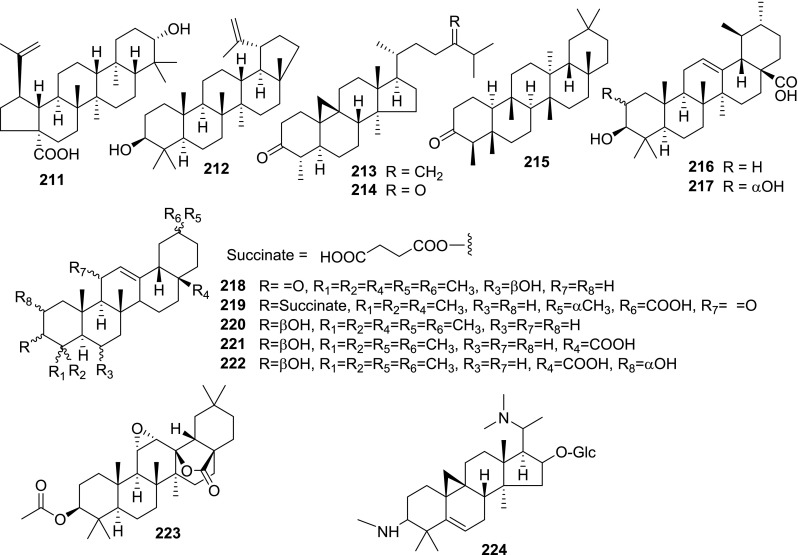
Triterpenoids **211**–**224** from *Solanum*

Fourteen triterpenes (**211**–**224)** were identified in *Solanum* spp. (Figure 4), with lupeol (**212**) from *S. cathayanum* [472, 473, 477]*, S. schimperianum* [278], *S. spirale* [297] and ursolic acid (**216**) from *S. lyratum* [197], *S. torvum* [463] and *S. xanthocarpum* [427], as the major ones. Six triterpenes **216**–**217** and **221**–**224** were reported from the aerial parts of *S. torvum* [314, 463]. Two cycloartane triterpenoids, cycloeucalenone (**213**) and 24-oxo-31-norcycloartanone (**214**) are the main constituents of *S. cernuum* leaves [107]. Daturaolone (**218**) was isolated for the first time from *S. arundo* [65].

*Solanum* triterpenes have indicated to possess anticancer properties. For instance, **213** presented significant activity against KB-Oral cavity cancer (IC_50_ = 26.73 μgmL) [297], while **213** exhibited selective activity against lung tumor cell line (NCIH460). The anti-nociceptive activity observed for **213** and **214** was found to be related to the inhibition of different mediators involved in inflammation and nociceptive process. Both compounds decreased cyclooxygenase 2 (COX-2) protein expression, although only **214** reached a significant response (*P* < 0.05 vs control) [107].

### Diterpenes

**Fig. 5:**
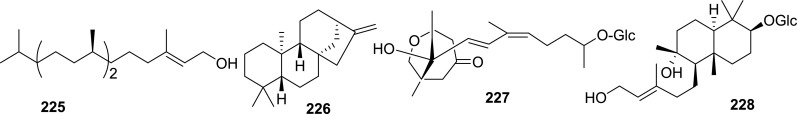
Diterpenes **225**–**228** from *Solanum*

Four diterpenes, e.g., phytol (**225**) from *S. pseudocapsicum* [263], kaur-16-ene (**226**) from *S. aculeastrum* [11], solanerioside A (**227**) from *S. erianthum* [138], and tricalysioside U (**228**) from *S. violaceum* [392] were reported from *Solanum* spp. (Figure 5). Solanerioside A (**227**) was the first example of a diterpenoid glucoside featuring a 14, 15-dinor-cyclophytane scaffold [138].

### Sesquiterpenes

**Fig. 6:**
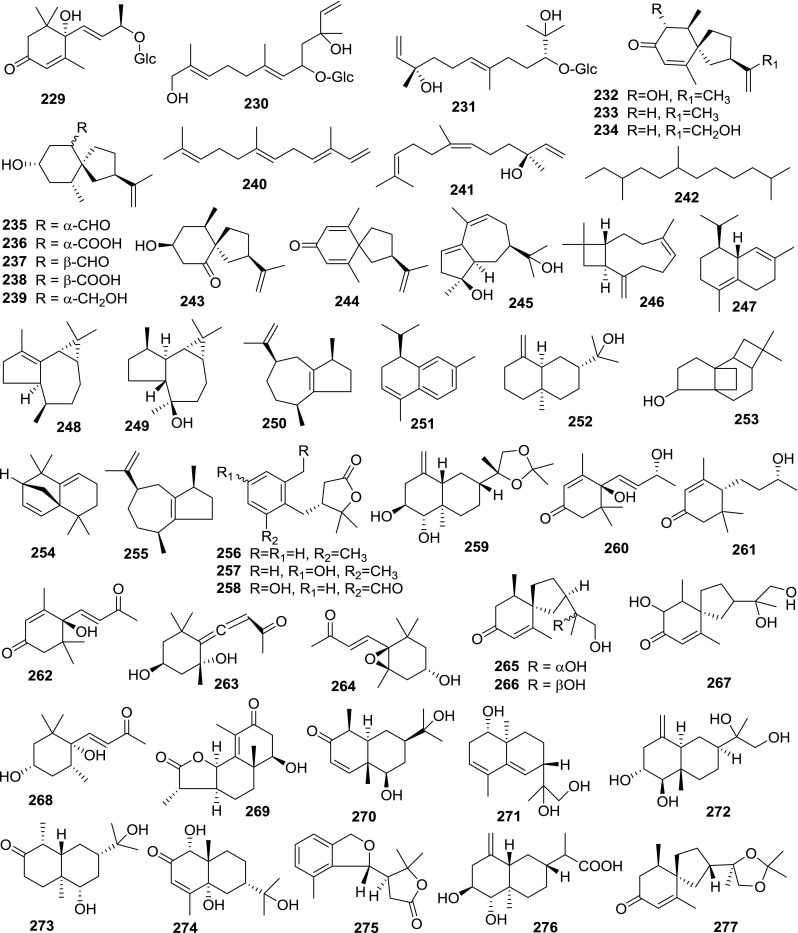

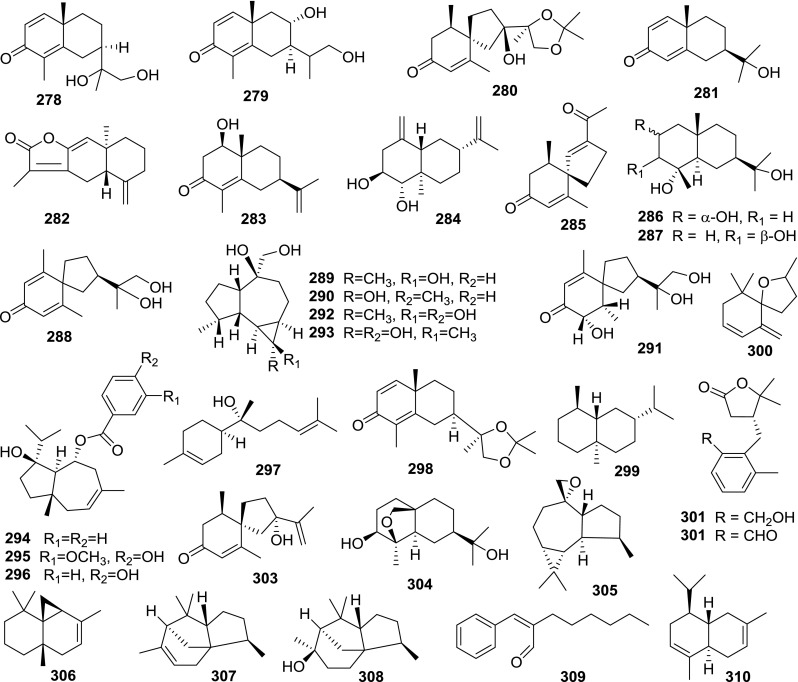
Sesquiterpenes **229**–**310** from *Solanum*

Sesquiterpenes, **229**–**310**, have been characterized from *Solanum* spp. (Figure 6). Majority of these compounds, **260**–**282**, were from *S. lyratum* [196, 197, 199, 200, 484–486] and *S. septemlobum* [281, 482, 483]. Likewise, **283**–**285** and **298**–**303** were reported from *S. septemlobum* [281, 482, 483]. Compounds **229**–**231** and **245**–**255** were isolated from the leaves and fruits of S*. erianthum* [138, 481], while **286**–**293** were from the roots of *S. torvum* [487]. Compounds **236**–**239** were isolated from the roots of *S. aethiopicum* [29], while **240**–**242** were obtained from the leaves of *S. aculeastrum* [11]. The fruits of *S. betaceum* yielded compounds **306**–**310** [77].

The bioactivities notedly displayed by sesquiterpenes include anticancer [197–200, 281, 484] and antifungal [3]. 3-*β*-Hydroxysolavetivone (**232**), solavetivone (**233**) and lubimin (**235**) from the roots of *S. abutiloides* exhibited anti-fungal activities against *Fusarium oxysporum* f. sp. Melongenae [3]. The eudesmane-type, solajiangxin D (**276**), and vetispirane-type, solajiangxin E (**277**) from *S. lyratum* demonstrated crucial cytotoxicities (ED_50_ = 2.1–3.7 μg/mL) against three human cancer lines (P-388, HONE-1, and HT-29) [200]. Solajiangxin B (**258**), A (**274**) and C (**275**) from the whole plant of *S. lyratum* [198] and Septemlobin D (**259**), and 11,12-*O*-isopropylidene solajiangxin F (**298**) [483] also showed significant cytotoxicities (ED_50_ = 1.9–3.7, and 3.0–7.3 μM, resp.) against these three cancer cell lines. Lyratol D (**257**), blumenol A (**260**), dehydrovomifoliol (**262**) and lyratol C (**272**) from the whole plant of *S. lyratum* displayed critical cytotoxic activities against HONE-1 nasopharyngeal, KB oral epidermoid carcinoma, and HT29 colorectal carcinoma cells (IC_50_ = 3.7–8.1 μM) [199].

Eudesmane-related sesquiterpenes, septemlobins A (**301**) and B (**302**) and vetispirane-type, septemlobin C (**303**) exhibited significant cytotoxicities against three cancer cell lines (P-388, HONE-1, and HT-29) (IC_50_ = 3.8–7.5 mΜ) [281].

### Monoterpenes

**Fig. 7:**
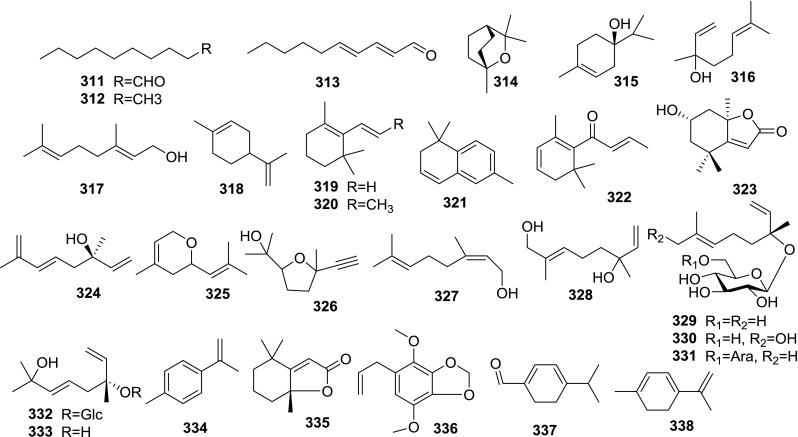
Monoterpenes **311**–**338** from *Solanum*

Twenty-eight monoterpenes (**311**–**338**) have been characterized from *Solanum* spp. (Fig. 7), with *β*-Ionone (**320**) reported from *S. aculeastrum* [11]*, S. pseudocapsicum* [263] and *S. betaceum* [77], and loliolide (**323**) obtained from *S. erianthum* [137], *S. americanum* [49] and *S. pseudocapsicum* [263], as dominant monoterpenes. Majority of the compounds, **316**-**318** and **324**–**333** [468, 489–492], were obtained from the fruits of *S. vestissimum*. Hotrienol (**324**), with very sweet and flowery flavor is a well-known constituent of the leaf oil of *Cinnamomum camphora*. It has also been found in a large number of other natural tissues, such as tea, grapes, wines passion fruit, elderberry flowers, *Achillea ligustica* and papaya fruit [468]. Seven monoterpenes, **311**–**313** and **319**–**322** were reported from the leaves of *S. aculeastrum* [11], and glycosides **329**–**332** were the aroma precursors in *S. vestissimum* fruit peelings [468, 492].

### Flavonoids

**Fig. 8:**
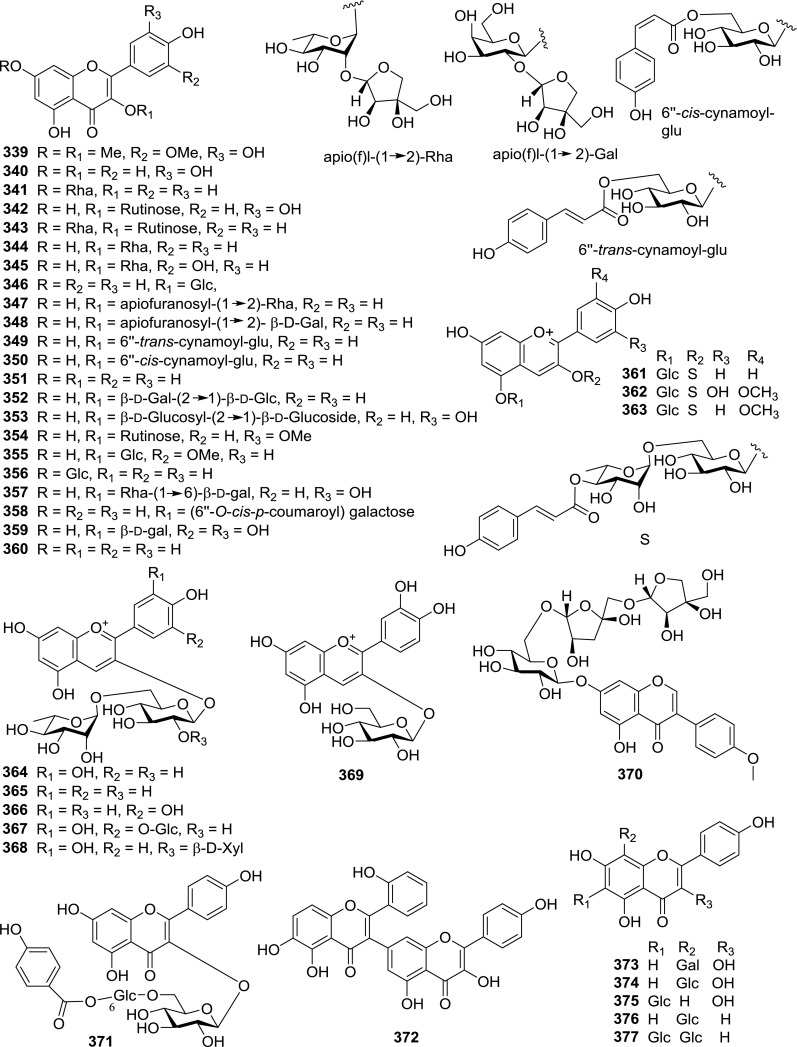

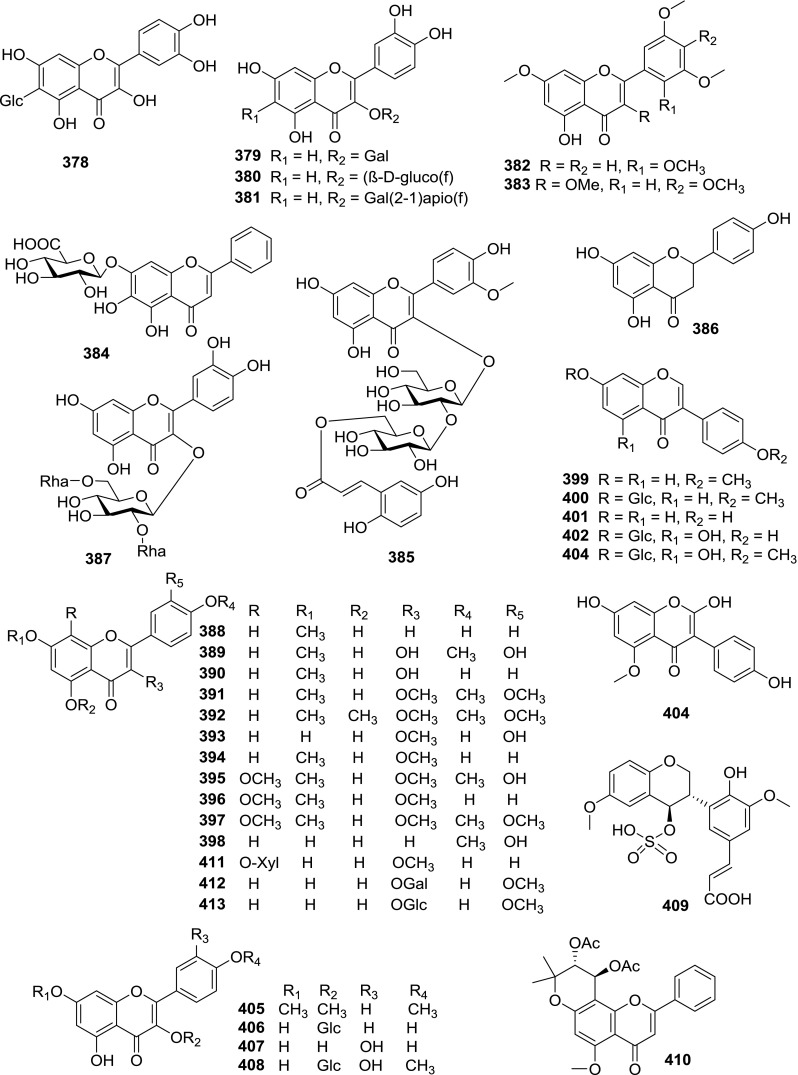
Flavonoids **339**–**413** from *Solanum*

Seventy-two flavonoids **339**-**413** have been identified in the genus *Solanum* (Fig. 8), with quercetin (**340**) and kaempferol (**351**) as the primary flavonoids. Several glycosylated flavonoids, e.g., afzelin (**344**), astragalin (**346**), kaempferol 3-*O*-[apiofuranosyl-(1 → 2)]- *α*-rhamnoside (**347**) and -*β*-galactoside (**348**) from *S. cernuum* [501], and camelliaside C (**352**) from *S. erianthum* [137] were obtained. Five kaempferol derivatives **373**–**377** were reported from *S. elaeagnifolium* [502]. Moreover, three anthocyanins **361**–**363** were isolated from the red and purple tubers of *S. tuberosum* [508], while five anthocyanin rutinosides **364**–**368** were reported from the fruits of *S. betaceum* [75, 76]. Anthocyanins are the largest group of water-soluble pigments in the plant kingdom. They are responsible for most red and blue colours in fruits, vegetables, and have been used in the food industry as pigments, owing to their bright attractive colours, high water solubility and associated health benefits [76]. In addition, diverse flavonoids, such as **388**–**397** from *S. jabrense* [167] and *S. palodusum* [513] and **399**–**403** from *S. lyratum* [514] were reported.

Flavonoids of *Solanum* have displayed various biactivities e.g., anticancer [31, 75, 76, 503], anti-depressant and antiviral [322, 332] and hepatoprotective [502] characteristics. Compound **373** exhibited significant hepatoprotective and curative effects against histopathological and histochemical damage induced by paracetamol in liver [502], while **349** and **371** displayed cytotoxicity against breast MCF7 and liver HPG2 cancer cell lines [503].

Compound **340** and rutin (**342**) indicated potent and concentration-dependent free radical-scavenging activity [45]. They also inhibited peroxidation of cerebral and hepatic lipids subjected to iron oxidative assault. Compound **340** induced in vitro antiproliferative and apoptotic activities on Jurkat cells (IC_50_ = 11.77 ± 2.4 mg/mL) [23], while **364**-**367** showed antioxidant activities [75]. Torvanol A (**409**) from the roots of *S. torvum* exhibited antidepressant, anxiolytic and adaptogenic effects [316], as well as anti-HSV-1 activity (IC_50_ = 9.6 μgmL) [322].

### Lignans

Lignans, widely distributed in the plant kingdom, are a family of secondary metabolites produced by oxidative dimerization of two phenylpropanoid units. Although their molecular scaffold consists only of two phenylpropane (C6–C3) units, lignans exhibit an enormous structural diversity originating from various linkage patterns of these phenylpropane units. As the C-8–C-3′/C-7–O–C-4′ linked lignans containing two chiral centers (C-7 and C-8) comprise the core of 2, 3-dihydrobenzo[b]furan [480].

**Fig. 9:**
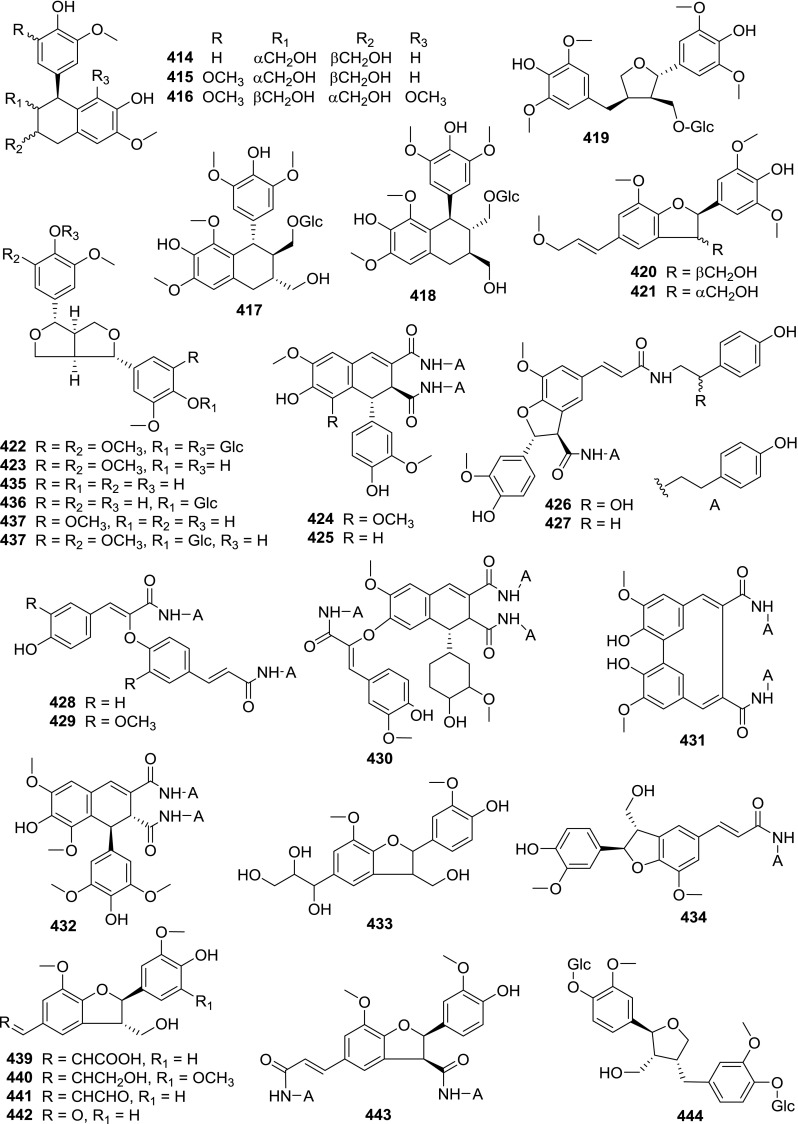
Lignans **414**–**444** from *Solanum*

Lignans are rare in the genus *Solanum* [79], with only 31 compounds (**414**–**444**) having been isolated (Fig. 9). Compounds **414**–**419** were obtained from the stems of *S. buddleifolium* [79], while **424**–**432**, **434** and **442** were isolated from the roots of *S. melongena* [208–210]. Several neo-lignans, sisymbrifolin (**433**) from the fruits of *S. sisimbriifolium* [519], ficusal (**442**) from the roots of *S. melongena* [209], glycosmisic acid (**439**), simulanol (**440**) and balanophonin (**443**) from the whole plant of *S. surattense* [518] were identified. A pair of new C-8–C-3′/C-7–O–C-4′ linked neolignan enantiomers, **420** and **421**, were isolated from the stems of *S. erianthum* [480]. Lignanamides **424**–**432** and **434** were obtained from the roots of *S. melongena* [210].

Among lignans from the genus *Solanum*, only lignanamides (**425**–**432**) were reported with bioactivities. They displayed anti-inflammatory activities by inhibition of nitric oxide production in lipopoly-saccharide-induced RAW 264.7 macrophages (IC_50_ = 16.2 to 58.5 μM) [210].

### Other Alkaloids

**Fig. 10:**
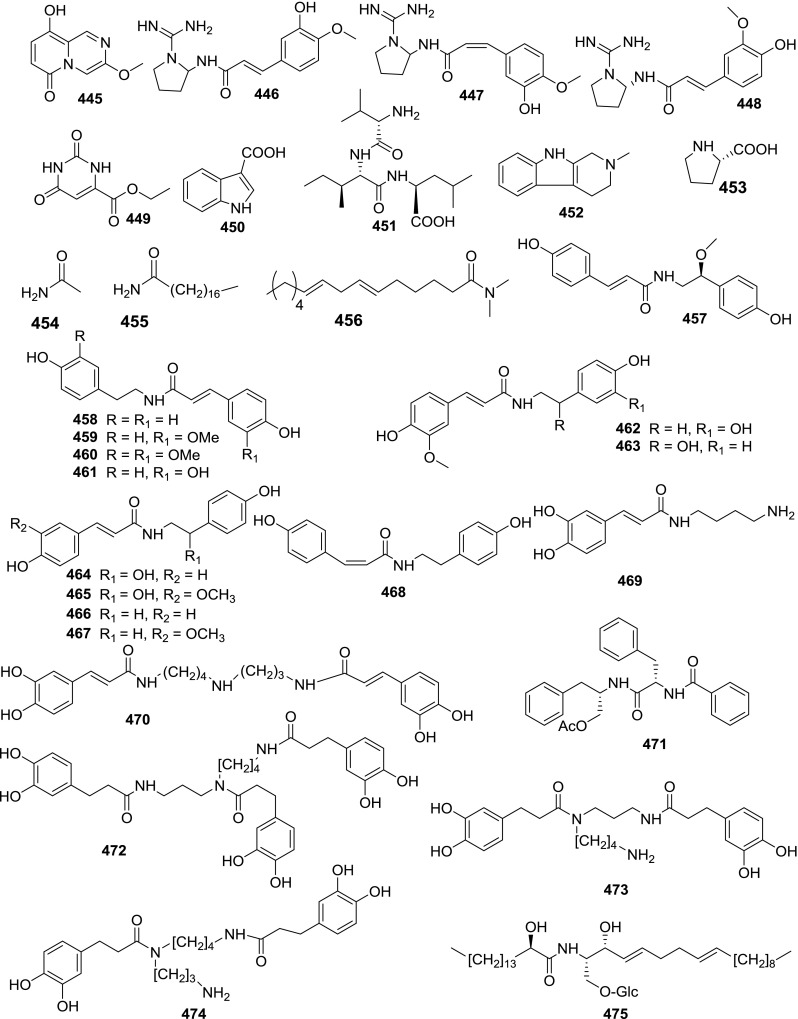
Other alkaloids **445**–**475** from *Solanum*

The alkaloids have a natural (2-aminopyrrolidin-1-yl) carboxamidine alkaloidal base acylated with isoferulic (3-hydroxy-4-methoxycinnamic) acid with Z and E configurations, resp. [111]. Thirty-one alkaloids **445**–**475** have been isolated from *Solanum* spp. (Fig. 10), comprising types of cyclic guanidine alkaloids, e.g., cernumidine (**446**) and isocernumidine (**447**) from the leaves of *S. cernuum* [109, 111, 112]. Bioactive long chain amides, **454**–**456**, exhibiting antimicrobial activity against *Escherichia coli* and *Candida albicans* were isolated from aerial parts of *S. schimperianum* [277]. Compounds **472**–**474** were obtained from *S. sessiliflorum* [525].

Antidiabetic activity was illustrated by *Solanum* alkaloids [49, 209]. Four amides, *N*-*trans*-*p*-coumaroyl -octopamine (**464**) and -tyramine (**466**), and *N*-*trans*-*p*-feruloyl -octopamine (**465**) and -tyramine (**467**) exhibited antidiabetic properties by enhancing α-glucosidase inhibitory activity in a study involving dual high-resolution *α*-glucosidaseradical scavenging inhibition profiling [35]. Moreover, **459**, **466** and **468** demonstrated possession of inhibitory activity against *α*-glucosidase (IC_50_ = 500.6, 5.3 and 46.3 μM, resp.) [209].

### Sterols

**Fig. 11:**
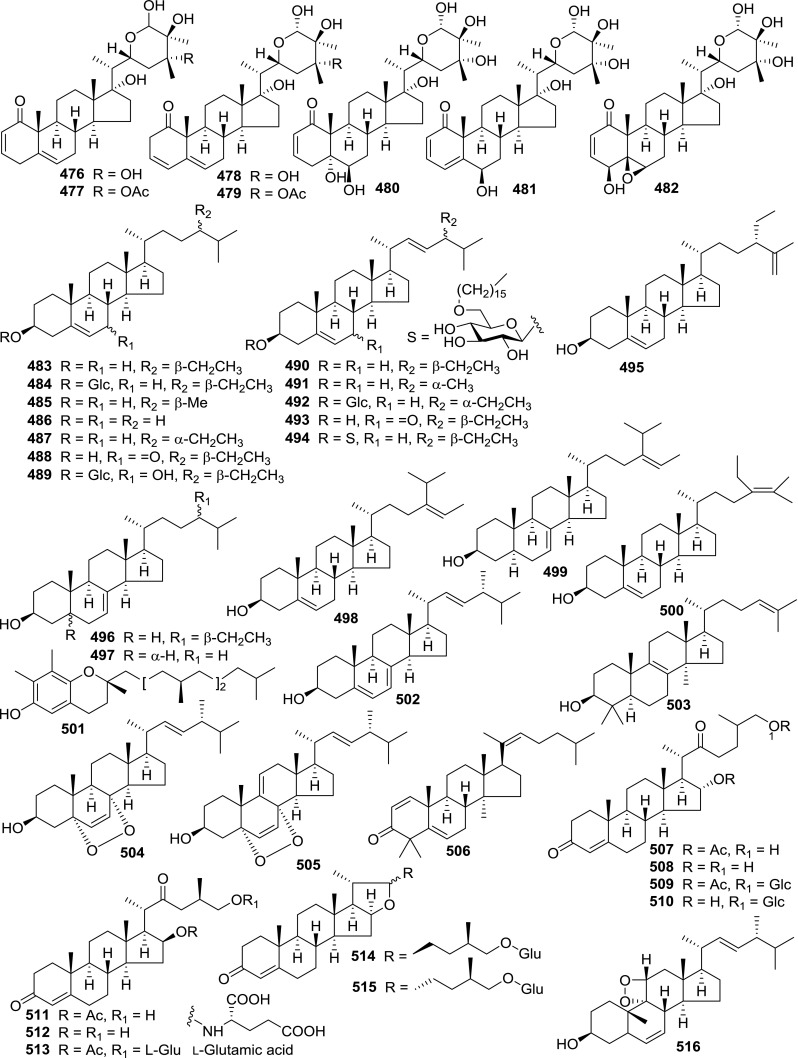

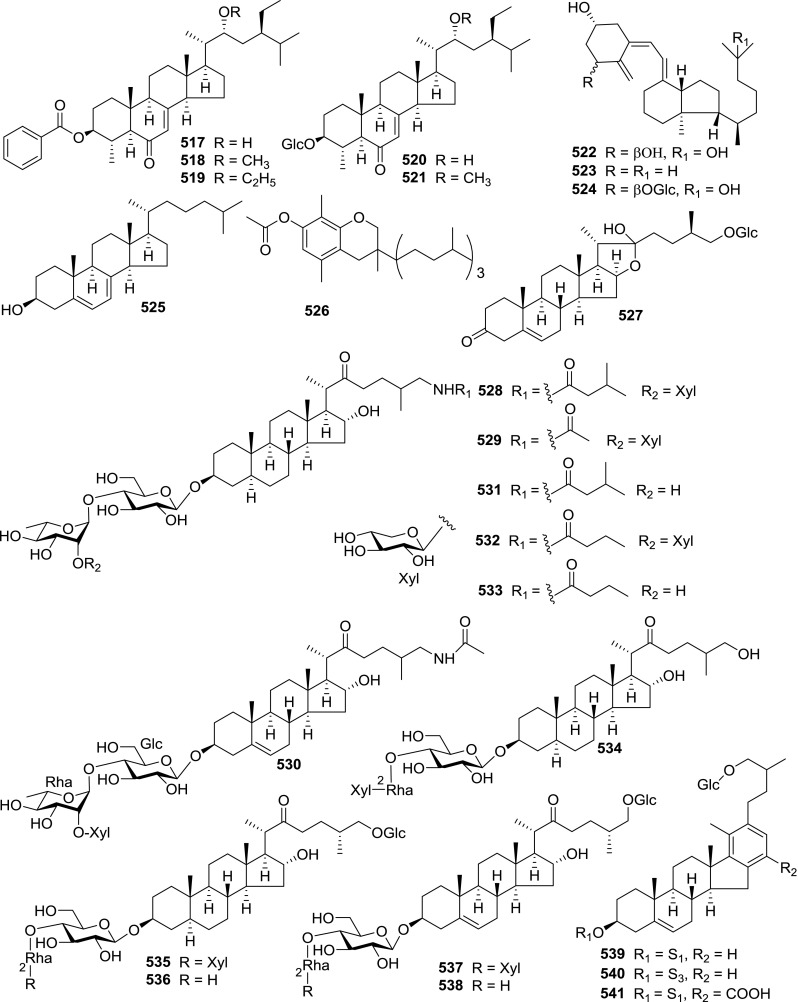
Sterols **476**–**541** from *Solanum*

Sixty-six sterols (**476**–**541**) were obtained from the genus *Solanum* (Fig. 11), with *β*-sitosterol (**483**), daucosterol (**484**) and stigmasterol (**485**) as the main sterol constituents. Clistol G (**476**) and capsisteroids A-F (**477**–**482**) were obtained from the leaves of *S. capsicoides* [85], tumacones A (**507**) and B (**508**) and tumacosides A (**509**) and B (**510**) were from the leaves of *S. nudum* [242–247], carpesterol (**517**) was isolated from the seeds of *S. capsicoides* [86], and its derivatives (**518**–**521**) were reported from the fruits of *S. xanthocarpum* [401]. From the seeds of *S. elaeagnifolium*, **491**, **495**, **496** and **498** were yielded [134]. Additionally, two 26-aminochole- stane-type glycosides, abutilosides A (**528**) and B (**529**), and five 26-hydroxycholestane-type glycosides, abutilosides C-G (**534**–**538**), were isolated from the fresh roots of *S. abutiloides* [5–9]. These compounds are important intermediates in the biogenesis of steroidal alkaloids [5].

Sterols in *Solanum* have indicated possession of anticancer [86], antifungal [401], and antiplasmodial [242, 245, 247] features. For instance, **509** and **510** displayed in vitro antimalarial activity against *P. falciparum* chloroquine-resistant FCB-1 strain (IC_50_ = 27 and 16 μM) [247]. Compounds **511**–**515** from aerial parts of *S. nudum* demonstrated antiplasmodial activity on hepatic trophozoites of *P. vivax*. All the steroids reduced the number of hepatic *P. vivax* trophozoites. Among them, **506** and **512** reduced the number of hepatic trophozoites by 47and 39resp. [245]. Compound **517** produced antiproliferative activity in glioma (U251), breast (MCF-7), kidney (786-0), ovary (OVCAR-03), and K562 cell lineages [86]. In addition, **505**–**509** displayed antifungal activity by inhibiting radial growth of *A. niger* and *T. viride* [401].

### Phenolic Compounds

**Fig. 12:**
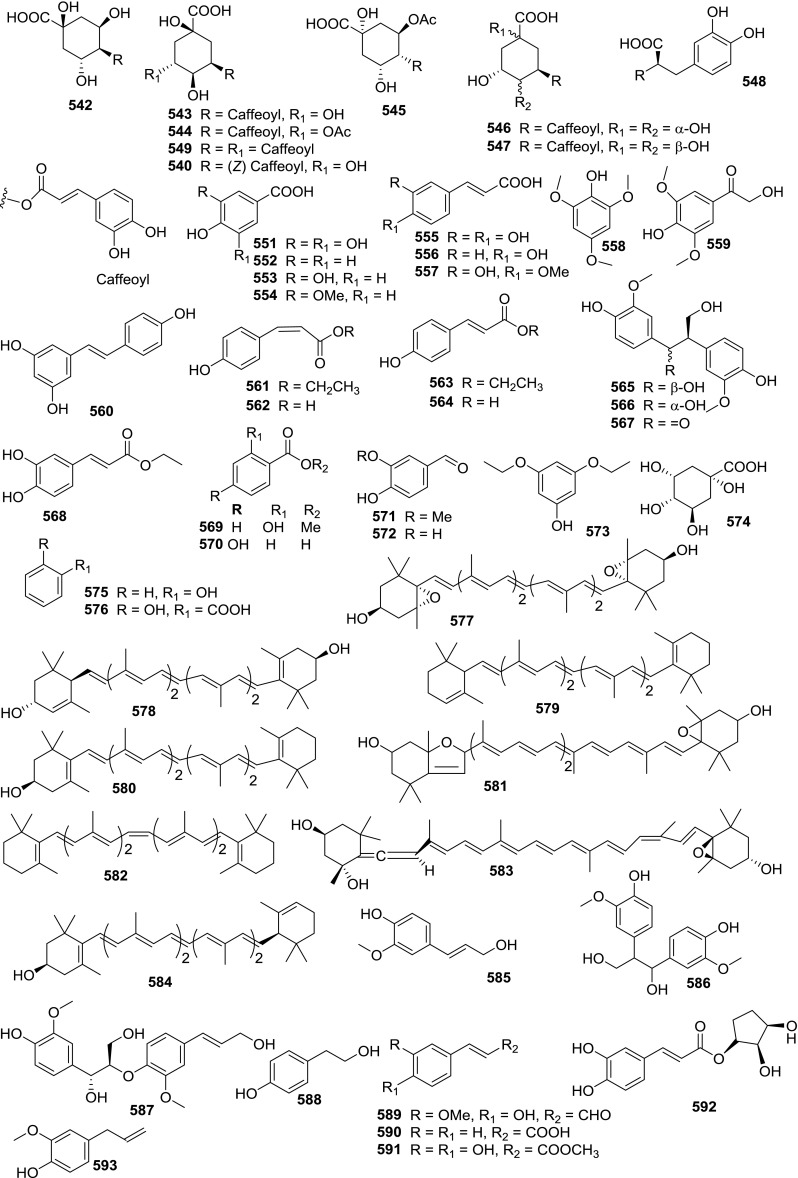
Phenolic compounds **542**–**593** from *Solanum*

Fifty-two phenolic compounds (**542**–**593**) were recorded from *Solanum* (Fig. 12). The fruits of *S. crinitum* have yielded **552**, **561**–**564** [122]. Aerial parts of *S. torvum* indicated a great wealth of phenolic compunds, e.g. **558**–**559**, **576**, **591**–**593** [315, 320, 335–337, 521, 524, 533]. The highest numbers of phenols, **542**–**546**, **549**–**540**, **552, 555** and **589** were reported from stems of *S. melongena* [205] while **574**–**575** and **577**–**584** were mentioned from the fruits *S. sessiliflorum* [525].

Phenolic compounds in *Solanum* have displayed antibacterial [297, 320, 335–337, 524], anticancer [31], anti- diabetic [297, 320, 335–337, 524] and antihypertensive [521] activities. Chlorogenic acid (**546**) (21.90 ± 0.02 mgg), gallic acid (**551**) (17.54 ± 0.04 mgg) and caffeic acid (**555**) (16.64 ± 0.01 mgg) have indicated potent and concentration-dependent DPPH radical-scavenging activity (IC_50_ = 275.03 ± 7.8 μg/mL) [31], and **551** and **555** reportedly have great potentials as natural source of antidiabetic and antioxidant drug [336]. *trans*-Cinnamic acid (**590**) showed antibacterial activities (MIC = 250 μg/mL) against *Staphylococcus aureus* [297], and antimycobacterial activities (inhibition zone = 0–22 mm) against *Proteus vulgaris, Klebsiella pneumoniae* (ESBL-), *M. tuberculosis* (H^37^Rv) and *M. tuberculosis* (Rifampin) [320]. Methyl caffeate (**591**) not only significantly reduced the cell proliferation, but also increased formation of fragmented DNA and apoptotic body in MCF-7 cells. In this study, Bcl-2, Bax, Bid, p53, caspase-3, PARP and cytochrome c release were detected by western blot analyses [474]. The effects of oral administration of **591** (10, 20 and 40 mgkg) in streptozotocin induced diabetic rats, including body weight, fasting blood glucose, plasma insulin, hemoglobin, glycated hemoglobin, total protein, hepatic glycogen and carbohydrate metabolism enzymes have been studied for 28 days. At 40 mgkg, the compound significantly prevented the increase in blood glucose level after glucose administration at 60 min in comparison to the hyperglycemic control group. It also produced remarkable reductions in blood glucose and increased body weight in streptozotocin induced diabetic rats [335]. Takahashi et al. further established that **591** has a most favorable structure for both sucrase and maltase inhibition against sucrose and that its moderate inhibitory action against alpha-glucosidase provides a prospect for antidiabetic usage of *S. torvum* fruit [337].

### Coumarins and Coumestans

**Fig. 13:**
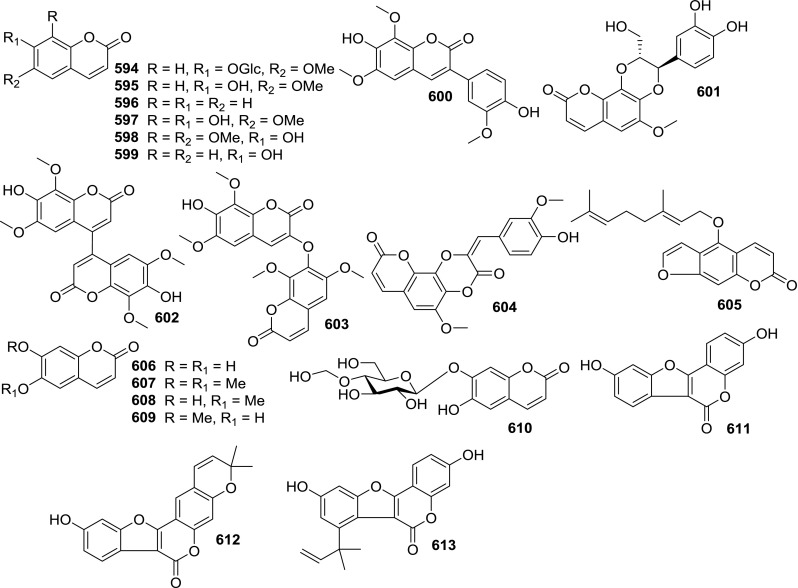
Coumarins and coumestans **594**–**613** from *Solanum*

Seventeen coumarins **594**–**610** and three coumastans **611**–**613** were isolated from *Solanum* spp. (Fig. 13). The seeds of *S. indicum* yielded the highest number of coumarins **597**–**598** and **600**–**604** [535, 536], while coumestans **611**–**613** were from the whole plant of *S. lyratum* [88]. Scopolin (**594**), scopoletin (**595**) and coumarin (**596**) are the main coumarins in *Solanum*. Compounds **611**–**613** showed in vitro anti-inflammatory activities with IC_50_ values in the range of 6.3–9.1 μM [88].

### Coumarinolignoids

**Fig. 14:**
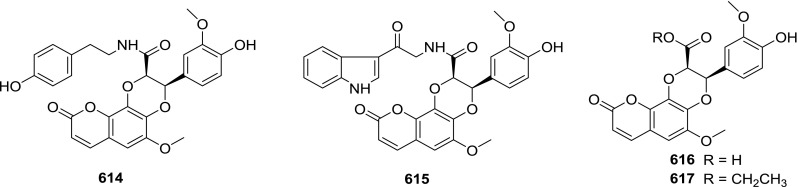
Coumarinolignoids **614**–**617** from *Solanum*

Four coumarinolignoids known as indicumines A–D (**614**–**617)** were obtained from the seeds of *S. indicum* [535] (Fig. 14). Coumarinolignoids, including cleomiscosins, aquillochins and malloapelins, are unique and rare in nature. Coumarinolignoids of the cleomiscosins type bearing cleomiscosins A–D, 8-*epi*-cleomiscosin A, and malloapeli A functionalities have been identified in a few genera, including *Cleome viscosa*, *Mallotus apelta*, and *Rhododendron collettianum*. The compounds with such functionalities, especially cleomiscosins A–C and 8-*epi*-cleomiscosin A, which contributed to biological activities, have been reported with hepatoprotective and tyrosinase inhibition activities [535].

### Fatty Acids and Esters

**Fig. 15:**
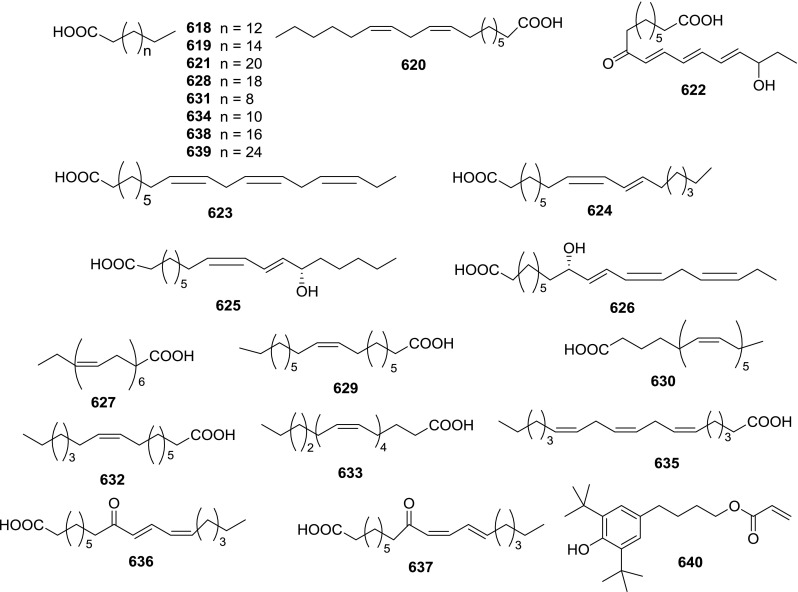
Fatty acids and esters **618**–**640** from *Solanum*

Nine saturated (**618**–**619**, **621**, **627**–**628**, **631**, **634**, **638**–**639**) and 13 unsaturated (**620**, **622**–**626**, **629**, **630**, **632**, **633**, **635**–**637, 640**) fatty acids were reported from *Solanum* (Fig. 15). The whole plant of *S. glabratum* has yielded the highest number of fatty acid and esters (**627**–**635**) in *Solanum* spp. [140]. Hexadecanoic acid (**618**), notably the major fatty acid component in *Solanum*, was isolated from aerial parts of *S. aculeastrum* [11] *S. vestissimum* [489] and *S. villosum* [434, 479].

### Others

**Fig. 16:**
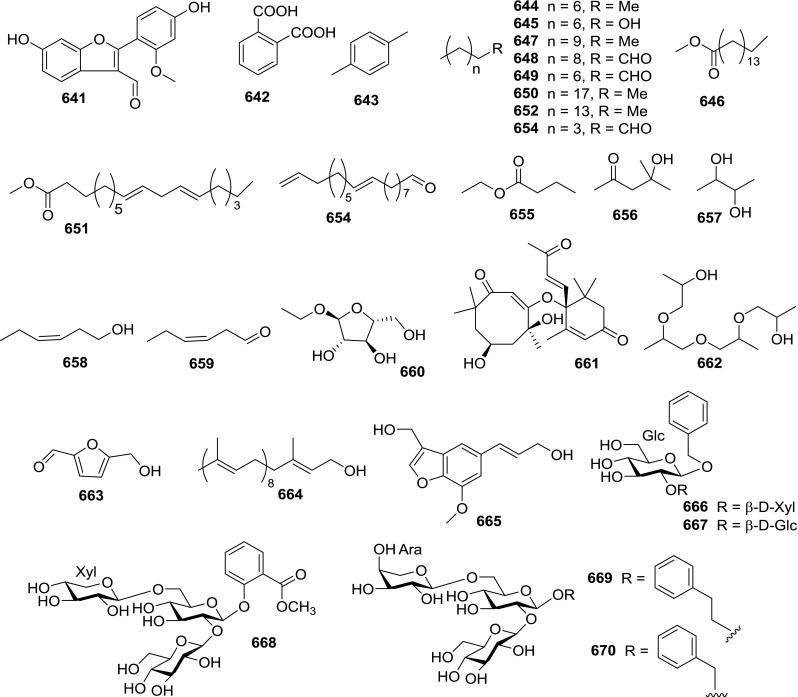
Other compounds **641**–**670** from *Solanum*

Thirty other kinds of compounds (**641**–**670**) were also obtained from *Solanum* spp. (Fig. 16). Most of them, **642**–**653**, were from the leaves of *S. aculeastrum* [11] and **654**–**659** were yielded from the fruits of *S. betaceum* [78]. An aldehyde puerariafuran (**641**) and a cyclic eight-membered *α*,*β*-unsataturated ketone, solalyratin B (**661**) were isolated from the whole plant of *S. lyratum* [88]. Compounds **641** and **661** showed in vitro anti-inflammatory activities, with IC_50_ values in the range 6.3–9.1 μM [88]. Also presented here are two furans, ethyl-*α*-D-arabinofuranoside (**660**) from the whole plant of *S. lyratum* and 5-hydroxymethyl furfural (**663**) from the stems of *S. torvum* [533]. Five aromatic glycosides (**666**–**670**) were also isolated from the aerial part of *S. incanum* [494] and the fruit of *S. lycopersicum* [511].

## Conclusion and Future Prospects

From 1990 to 2017, phytochemical studies on the 65 *Solanum* species have yielded at least 670 compounds (134 steroidal saponins, 63 steroidal alkaloids, 13 pregnane glycosides, 128 terpenes, 75 flavonoids, 31 lignans, 31 alkaloids, 66 steroids, 52 phenolic compounds, 20 coumarins and coumestans, 4 coumarinolignoids, 23 fatty acids and esters, and 30 other types of compounds).

Pharmacological studies on *Solanum* genus have focused on antioxidants and anticancer activities. A total of 17 species (fruits of *S. aculeastrum, S. americanum, S. muricatum, S. sessiliflorum* and *S. spirale,* seeds of *S. capsicoides,* the stems of *S. cathayanum* and *S. tuberosum,* the roots of *S. diphyllum,* aerial parts of *S. surattense* and *S. torvum* and the whole plant parts of *S. aethiopicum, S. nigrum, S. anguivi, S. septemlobum, S. violaceum* and *S. xanthocarpum*) have been explored for anticancer activities and have exhibited significant results.

*S. xanthocarpum* has outstandingly demonstrated the most diverse pharmacological activities *e.g*. antioxidants and antitumor, anti-fungal, anti-bacterial, antileishmanial, mosquito larvicidal, molluscicidal, antidiabetic, asthmatic,hepatoprotective, diuretic, nephrotoxicity, antinociceptive, anti-psoriatic, and antiurolithiatic.

Steroidal alkaloids have been presented as being largely responsible for various pharmacological activities of *Solanum* species, *e.g*. antibacterial (**139**, **141** and **145**), anticonvulsant and CNS depressant (**145**), antidiabetic (**139**, **142** and **144**), anti-fungal (**145** and **174**), anti-inflammatory (**145**), antileishmanial (**139** and **142**), molluscicidal (**139** and **141**), nephrotoxicity (**168**), antioxidants and antitumor (**139**, **141**, **145**, **158**, **168** and **180**), antiprotozoa (**139** and **142**), schistosomicidal (**139** and **142**), spasmolytic (**190**) and anti-trypanosomal (**139**).

The genus *Solanum* seems to possess great potential, yet majority of the species remain unknown or scantily studied for the chemical constituents. It would be very necessary for the phytochemistry researchers to explore and investigate more of its species. The vast pharmacological activities envinced by many compounds from *Solanum* genus should attract the attention of the pharmacological community to determine their exact target sites, structure–activity relationships and other medicinal applications.

## Abbreviations

ABTS: 2,2′-Azino-bis(3-ethylbenzthiazoline-6-sulphonic acid)
CC_50_: Cytotoxic concentration of the extracts to cause death to 50% of host’s viable cells
CDDP: *cis*-Diamminedichloroplatinum
DPPH: 2,2-Diphenyl-1-picrylhydrazyl
EC50: Half maximal effective concentration
GABA: Neurotransmitter gamma-aminobutyric acid
HBV: Hepatitis B Virus
HSV-1: Herpes simplex virus type 1
IC50: Minimum inhibition concentration for inhibiting 50% of the pathogen
LD50: Dose required to kill half the members of a tested population after test duration
MIC: Minimum inhibitory concentration
MTT: 3-(4,5-dimethylthiazol-2-yl)-2,5-diphenyltetrazolium bromide
SAG: Superoxide anion generation
